# The role of high-amplitude bursts of high-gamma activity in naturalistic speech and music listening

**DOI:** 10.1162/IMAG.a.1348

**Published:** 2026-08-27

**Authors:** Matteo Neri, Claudio Runfola, Pierre Guilleminot, Noemie te Rietmolen, Pierpaolo Sorrentino, Daniele Schon, Benjamin Morillon, Giovanni Rabuffo

**Affiliations:** Aix Marseille Université, INSERM, INS, Institut de Neurosciences des Systèmes, Marseille, France; Aix Marseille Université, CNRS, INT, Institut de Neurosciences de la Timone, Marseille, France; Language and Computation in Neural Systems Group, Max Planck Institute for Psycholinguistics, Nijmegen, Netherlands

**Keywords:** intracranial EEG, naturalistic stimuli, speech, music, high-gamma bursts, neuronal avalanches, temporal response function

## Abstract

A central challenge in systems neuroscience is to understand how distributed brain networks organize activity during naturalistic cognition. Here, we investigate whether transient, high-amplitude bursts of high-gamma activity, referred to as neuronal avalanches, provide a compact and functionally informative description of large-scale neural dynamics during auditory processing. Using intracranial stereotactic intracranial electroencephalography (sEEG) recordings from epileptic patients, we analyzed brain activity during speech listening, music listening, and rest. We show that high-amplitude bursts, defined as the top 1% of high-gamma activations, are strongly stimulus-driven: they synchronize across participants exposed to the same auditory input, exhibit condition-specific spatial topographies, and display distinct propagation patterns across cortical networks. Notably, although these events represent only a small fraction of the signal, they preserve substantial stimulus-related information. Temporal response function analyses reveal that avalanche activity reliably encodes both speech and music stimuli, even under extreme data sparsification. Together, these findings demonstrate that neuronal avalanches capture stimulus-relevant, large-scale neural dynamics and provide a computationally efficient framework for studying cognition in naturalistic settings.

## 1 Introduction

The brain is a complex system operating across multiple spatial and temporal scales. In order to decode brain functions, researchers analyze brain data across imaging modalities searching for regularities, such as patterned processes that might correlate with specific behaviors or cognitive abilities (Bressler & Menon, 2010; Park & Friston, 2013). Recurrent patterns can be observed, for instance, in the spectral analysis of brain oscillations (Buzsáki & Watson, 2012; Palva & Palva, 2007; te Rietmolen et al., 2024) or in the investigation of functional connectivity (Arbabshirani et al., 2014; Bassett & Sporns, 2017; Bullmore & Sporns, 2009; Mazzara et al., 2025). One ubiquitous finding, observed across imaging modalities and spatial and temporal scales, is the spontaneous occurrence of high-amplitude bursts recurring aperiodically in neural activity and propagating across the neuronal networks in cascade-like events (Beggs & Plenz, 2003; Neri, Angioielli, et al., 2025; J. M. Palva et al., 2013; Priesemann et al., 2013; Rabuffo et al., 2021; Shriki et al., 2013; Tagliazucchi et al., 2012). Historically, the interest in the study of these events originated from the observation that their statistics (e.g., the seemingly scale-free distribution of event duration and size) drew parallels with natural phenomena like sandpile avalanches and forest fires (Bak et al., 1988). For this reason, they were termed *neuronal avalanches*.

Neuronal avalanches have been hypothesized to be the manifestation of a critical state of neural dynamics, corresponding to neural activity occupying a sweet spot at the edge of two different regimes of organization (e.g., synchronous and asynchronous (Buendía et al., 2021; di Santo et al., 2018)). Brain criticality was proposed as a general organizing principle for the brain (Plenz et al., 2021) and has been hypothesized to play a crucial role in information processing (Shew et al., 2011), adaptive behavior (Bansal et al., 2021; J. M. Palva et al., 2013), and robustness of brain networks (Pajevic & Plenz, 2009; Zhou et al., 2021). While the brain criticality hypothesis remains debated (Beggs & Timme, 2012), evidence shows that neuronal avalanches are a valid marker of physiological brain states beyond the criticality assumption (Turkheimer et al., 2022). For instance, neuronal avalanche features differ across health and pathology (Agouram et al., 2025; Duma et al., 2023; Polverino et al., 2022; Rucco et al., 2020; Seshadri et al., 2018; Sorrentino, Rucco, et al., 2021) or across resting wakefulness and sleep states (Priesemann et al., 2013; Scarpetta et al., 2023).

As a whole, this literature suggests the relevance of focusing on the digital nature of high-amplitude bursts of brain activity as an alternative to considering the continuous nature of neural time series. However, most studies in humans have analyzed avalanches in task-free conditions (Arviv et al., 2019; Bansal et al., 2021). An open question hence remains about whether these kinds of phenomena index cognitive functions or purely reflect physiological states. Moreover, studying neuronal avalanches is particularly relevant in the context of naturalistic cognition, such as speech and music processing, because these stimuli are inherently structured across multiple temporal and spatial scales. Speech and music contain features ranging from fast acoustic fluctuations to slower prosodic, syntactic, and semantic patterns, requiring neural systems to operate over a wide dynamic range. Theoretical and empirical work has shown that neural dynamics close to criticality maximize dynamic range and sensitivity to inputs spanning multiple scales, while also supporting flexible coordination across distributed networks (Shew & Plenz, 2013). In this framework, avalanche-like activity provides a parsimonious description of how the brain may efficiently encode and integrate multiscale stimulus features during naturalistic perception.

Furthermore, recent literature has argued that applying analytical frameworks used in resting-state studies to cognitive task datasets could elucidate how cognition emerges from neuronal activities (Finn, 2021). Within this context, experiments based on naturalistic stimuli offer a convenient trade-off between overly specific task paradigms and non-specific resting-state studies (Dini et al., 2023; Hasson et al., 2005, 2010; Runfola et al., 2025; Sonkusare et al., 2019). The use of ecologically valid stimuli, both in terms of content and duration, makes it possible to study changes in neuronal avalanche properties in continuous brain activity induced by specific natural cognitive states (Corsi et al., 2022; Hamilton & Huth, 2020; Sonkusare et al., 2019). This allows investigating the hypothesis that avalanches are a neurophysiological correlate of cognitive processes and, as such, that their properties are modulated according to the stimulus at hand.

**Fig. 1.**
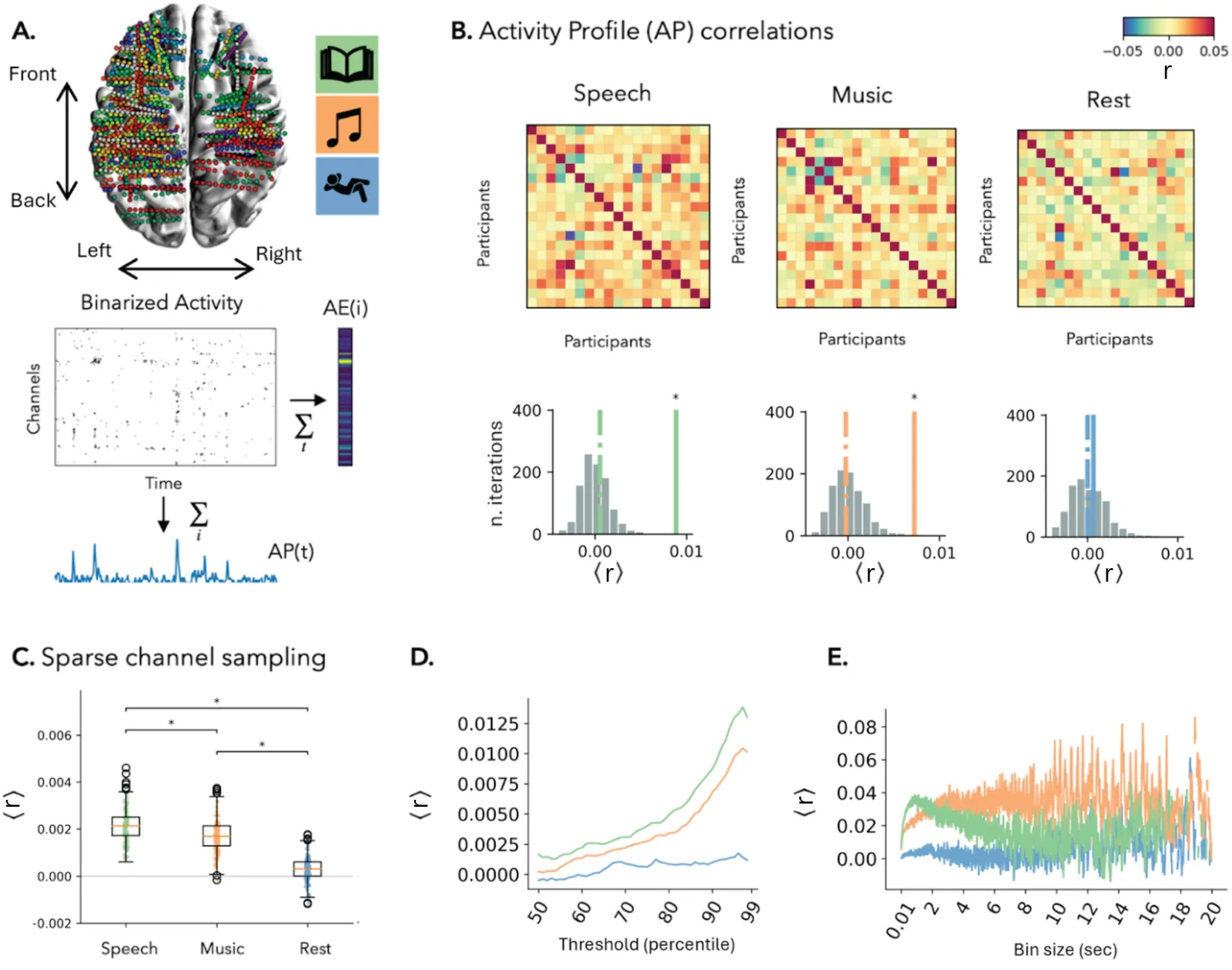
(A) **(top left)** Anatomical localization of the sEEG electrodes for each participant (N = 19); different colors correspond to different participants. **(top right)** The three natural conditions under study: speech, music, and rest. **(bottom)** Binarized HFa over time, for the different sEEG channels of a representative participant. The Activity Profile (APr) and the Activity Engagement (AE) are obtained by summing the binarized activity matrix across across channels and time, respectively. (B) **(top)** Inter-subject correlation (r) matrix for the three conditions. Each point of the matrix represents the correlation value between the APr of two participants. **(bottom)** Mean of the inter-subject correlation (colored line) compared to a null model distribution (see Methods; *p < 0.001). (C) Distribution of mean inter-subject correlation obtained by randomly selecting one channel per anatomical region (1000 iterations) in all the participants. The distributions for speech, music, and rest are compared using the Wilcoxon signed-rank test (*p < 0.001). The dotted line represents the result obtained when keeping for each patient instead of the highest, the lowest peaks. (D–E) Average inter-subject correlation computed by binarizing the data with (D) different thresholds and (E) bin sizes (see Methods). HFA, high-frequency activity; APr, Activity Profile, reflecting the spatial distribution of high-amplitude events; AE, Activity Engagement, reflecting the level of event participation across recording sites or regions; r, Pearson correlation coefficient, computed consistently with the other analysis of the paper using Pearson correlation.

To test this, we analyzed neuronal avalanches recorded in 19 pharmaco-resistant epileptic participants using stereotactic intracranial electroencephalography (sEEG) recordings, with channels distributed across the entire brain (Fig. 1A). Such recordings provide anatomically precise information about the functionally-selective engagement of neuronal populations at the millimeter scale as well as about their temporal dynamics at the millisecond scale. This setup is essential for accurately portraying the neurophysiological underpinning of a specific cognitive process (Martin et al., 2016; Mercier et al., 2022). In particular, we focused on high-frequency activity (HFa, 80–120 Hz), because it is an effective index of local neuronal processing and is highly correlated with local neuronal spiking activity (Ray & Maunsell, 2011). To establish ecologically-relevant stimulation conditions, participants listened to long and natural audio recordings of either speech or music and also underwent a control resting-state (rest) session (~10 minutes per condition).

Given our hypothesis, we expect that the neuronal avalanche timing and propagation across the brain is affected by the presence and the nature of the stimuli (Bansal et al., 2021). For each condition, we analyzed the temporal and topographic properties of neuronal avalanches, showing that these salient events synchronize across participants listening to the same auditory stimulus, revealing common neural patterns associated with processing of temporally structured musical and linguistic patterns. To further probe speech and music processing, we tracked the spatial propagation of avalanches, identifying the regions that participate in coordinated events and testing how music and speech modulate the spread of avalanche activity across the brain. Although avalanches comprised only the top 1% of the strongest activations, they carried substantial stimulus-related information: avalanche activity correlated significantly with stimulus predictions derived from temporal response functions. Moreover, recording sites with the highest decoding performance were also those most strongly engaged in avalanche activity. Overall, focusing on neuronal avalanches provided low-computational-cost access to new insights into brain function (Lannelongue & Inouye, 2023), supporting the view that avalanches are network-level correlates of cognitive processes that capture both distributed and region-specific functional properties.

## 2 Materials and Methods

### 2.1 Participants

Nineteen participants (10 females, mean age 30 years old, one 8-year-old patient, the others with age in the range 17–54 years old) with pharmacoresistant epilepsy participated in the study. All patients were French native speakers. Neuropsychological assessments, carried out before stereotactic intracranial EEG (sEEG) recordings, indicated that all participants had intact language functions and met the criteria for normal hearing. In none of them were the auditory areas identified as part of their epileptogenic zone, as per the rating of an experienced epileptologist. Recordings took place at the Hôpital de La Timone (Marseille, France). The study was approved by the Assistance Publique – Hôpitaux de Marseille (health data access portal registration number PADS 3ZSKYE).

### 2.2 Data acquisition

The sEEG signal was recorded using depth electrodes shafts of 0.8 mm diameter containing 10 to 15 electrode contacts (Dixi Medical or Alcis, Besançon, France). The contacts were 2 mm long and were separated from each other by 1.5 mm. The locations of the electrode implantations were determined solely on clinical grounds. Participants were included in the study if their implantation map covered at least partially the Heschl’s gyrus (left or right). The cohort consists of 13 unilateral implantations (10 left, 3 right) and 6 bilateral implantations, yielding a total of 271 electrodes and 3371 contacts (see Fig. 1A, Supplementary Figs. S3, S4, and S7C for electrodes localization).

The patients were recorded either in an insulated Faraday cage or in the bedroom. In the Faraday cage, participants sat comfortably in a chair, the room was sound attenuated, and data were recorded using a 256-channels amplifier (Brain Products), sampled at 1 kHz and high-pass filtered at 0.016 Hz. In the bedroom, data were recorded using a 256-channels Natus amplifier (Deltamed system), sampled at 512 Hz, and high-pass filtered at 0.16 Hz.

### 2.3 Experimental design

The participants passively listened to 10 minutes of storytelling (576.7 secs, La sorcière de la rue Mouffetard, ref Gripari P. 2004) and 10 minutes of music (580.36 secs, Reflejos del Sur, ref Oneness, 2006) embedded in 3 resting-state sessions (rest; each lasting 3.3 minutes). The order of the speech and music conditions was balanced across participants. In all our analyses, we concatenated the 3 resting-state sessions into a single 10-minute-long rest condition. In the Faraday cage, a sound Blaster X-Fi Xtreme Audio, an amplifier Yamaha P2040, and Yamaha loudspeakers (NS 10M) were used for sound presentation. In the bedroom, stimuli were presented using a Sennheiser HD 25 headphone set. Sound stimuli were presented with a 44.1 kHz sample rate and 16 bits resolution. Speech and music excerpts were presented at ~75 dBA.

### 2.4 Preprocessing

Contact data were converted offline to virtual channels using a bipolar montage approach (closest-neighbor contact reference) to increase spatial resolution and reduce passive volume diffusion from neighboring areas (Mercier et al., 2017). To precisely localize the channels, a procedure similar to the one used in the iELVis toolbox was applied (Groppe et al., 2017). Anatomical localization of the channels was further labeled with the Brainnetome Atlas (Supplementary Figs. S3, S4, and S7C; Fan et al., 2016).

Bipolar channels outside the brain were removed from the data (~3%). The remaining data were bandpass filtered between 0.1 and 250 Hz and, when necessary, a notch filter was additionally applied at 50 Hz and harmonics up to 200 Hz, to remove power line artifacts. Finally, the data were downsampled to 500 Hz.

### 2.5 High-frequency activity (HFa)

The amplitude of the high-frequency activity (HFa, 80–120 Hz) was obtained by computing—via the Hilbert transform—the analytic amplitude of four 10-Hz-wide sub-bands spanning from 80 to 120 Hz. Each sub-band was standardized by dividing it by its mean and, finally, all sub-bands were averaged together (Ossandón et al., 2012; Vidal et al., 2012). To further reduce the computational cost, the data were downsampled to 100 Hz.

### 2.6 Artifact rejection

Channels with a variance greater than 2*IQR (interquartile range, i.e., a non-parametric estimate of the standard deviation) were tagged as artifacted channels. Then, for the remaining channels, time-segments with activity greater than 5 standard deviations were considered to be artifacted and replaced with the mean of the signal. This prevented destroying the temporal structure of the data, which is necessary to evaluate the inter-subject correlations.

### 2.7 Neuronal avalanches

Neuronal avalanches were estimated by binarizing the z-scored HFa and selectively focusing on salient, above threshold (99 percentile), events in the neuronal dynamics (Fig. 1A). This approach captures higher-order neuronal dynamics while being a sparse code and hence being computationally efficient. Neuronal avalanches are then defined as consecutive above-threshold activations and correspond to collective network events occurring across subsets of recording channels. As a convention, an avalanche starts when at least one channel is ‘active’, and ends when no channels are above the threshold (Supplementary Fig. S1). We tested whether the observed neuronal avalanche durations and sizes followed power-law distributions (expected at criticality) by pooling the data across participants in the same condition (Supplementary Fig. S2). The distributions more closely matched a power-law than an exponential model (Supplementary Fig. S2) but were not statistically different than a lognormal distribution. Furthermore, results were variable across individual participants, possibly due to limited recording time and spatial sampling. We also calculated the branching ratio, finding values near 1 in all conditions suggesting near-critical dynamics (Supplementary Fig. S2). While we could not draw definitive conclusions on criticality, our main focus in this work was to characterize the spatial and temporal features of high-amplitude bursts of activity during speech, music, and rest. Since our binarization process aligns with traditional methods in neuronal avalanche research, throughout the manuscript we will use the term “neuronal avalanches” and “high-amplitude burst of activity” interchangeably.

### 2.8 Activity profile

The activity profile (APr) reflects the timing of neuronal avalanches, and is estimated as the sum of the binarized HFa across channels (Fig. 1A). It measures the number of channels participating in a neuronal avalanche at each time point. To this end, HFa signals $X_{i}\left(t\right)$ were first z-scored across time, as, $z_{i}\left(t\right)=\frac{X_{i}\left(t\right)-{\bar{X}}_{i}}{\sigma \left(X_{i}\right)}$; where $\bar{X}$ and σ(X) denote the mean and standard deviation of the signals, respectively. The binarization was then performed by dividing the data in bins of *m* time steps. If a bin contains at least one time-point in which the activity in absolute value is higher than the fixed threshold, the bin is assigned value 1, and 0 otherwise. The threshold was fixed as the 99th percentile of the (absolute value of) z-scored resting-state activity, and the same threshold was used for all conditions, that is, speech, music and rest. With this choice, binarization yields 99% of time points in which the high-gamma activity remains below threshold and only 1% of time points in which high-gamma activity exceeds the threshold. The threshold was measured separately for each participant as the 99th percentile of resting-state high-gamma activity, but then kept constant across conditions, ensuring a comparable number of activations across subjects and enabling a fair, direct comparison across conditions. Figure 1D shows that such a high threshold value guarantees optimal inter-subject Pearson correlation results, in line with the idea that brain dynamics is largely driven by rare activation events. Unless differently specified, we fixed *m* = 2 to guarantee high temporal resolution while reducing noise effects. Notice that the tuning of the bin size *m* can play an important role in detecting critical-like avalanches (Fontenele et al., 2023). To check for the stability of our results, in Figure 1E we computed the inter-subject correlation using different *m*, which provides indirect insights about the temporal scales of speech and music processing.

### 2.9 Activity profile correlations

The inter-subject correlation of the APr over time was obtained by computing the correlation between the APr of each pair of participants and averaging across all the pairs. To test whether inter-subject correlations are statistically significant, we generated null models by randomly shifting the APr of each participant before computing the inter-subject correlation. We iterated this process 1000 times and we compared the null-distributions of the inter-subject correlations with the observed inter-subject correlations. To establish significance, we computed p values as the proportion of iterations in which the correlation in the surrogate data was higher than the observed one. To test that inter-subject correlations are not exclusively driven by local interactions, that is, within channels situated in the same cortical regions, we sampled randomly one channel per atlas-based region in all the participants. We calculated the correlation between the APrs, computed using only the sampled recording sites. We iterated this process 1000 times and we established the significance of our results computing p values as the fraction of samples (in total 1000) in which a positive inter-subject correlation was measured. The distributions of the 1000 results are compared across conditions using the Wilcoxon signed-rank test (Fig. 1C).

### 2.10 Dynamic inter-subject correlation

To investigate the temporal evolution of the inter-subject correlation, we correlated the APr in a time-resolved way, using a sliding window approach. That is, we looked at the evolution of inter-subject correlation between APr across time windows of 150 time points (3 seconds). To maintain a good temporal resolution, we used a sliding step equal to the temporal resolution of the binarized time series, that is, 20 milliseconds. This way, we obtained, for each condition, a time series for the inter-subject correlation.

### 2.11 Activity engagement (AE)

In order to perform a topographic analysis of neuronal avalanches across speech, music, and rest conditions we defined the activity engagement (AE) as the sum over time of the binarized signals (Fig. 1A). Then, for each brain region i, we assessed the significance of the observed AE(i) by pairwise comparisons of the speech, music, and rest conditions.

Given any two conditions A and B, we defined a null-distribution of AENull (i) based on the null-hypothesis that AE_A_(i) and AE_B_(i) are not different from each other. According to this, starting from the i-th binary signal of time length n in conditions A and B, we defined a new binary sequence by randomly selecting n time bins from the joint A and B signals. The sum of this new sequence defines a “null” AE for the i-th channel. This process can be repeated a large number of times to build a null-distribution of AENull (i) values. For a large number of iterations this distribution approaches a hypergeometric distribution with mean k = (AE_A_(i) + AE_B_(i))/2 and variance k(n − k)/(2n− 1). This allowed us to efficiently perform statistical testing by comparing AE in two conditions against the expected theoretical distribution, avoiding computationally expensive iterations. Hence, for each region *i* we compared the AE_music(or speech)_(i) computed during music (or speech) listening, with AE_rest_(i). The difference AEmusic(i) – AErest(i) was evaluated against a null distribution corresponding to the hypothesis that the two AE values were drawn from the same recording session. This comparison yielded a p-value for each region. Channels for which this difference was significant (p < 0.05, Bonferroni corrected for the number of channels) were labeled as “significant-AE channels for music (or speech)” (Supplementary Fig. S9A).

We also utilized this approach to compare the AEs for speech and music computed exclusively over the time steps belonging to the windows of times in which the intersubject correlation for speech and music was higher than the maximum observed in resting state (Supplementary Fig. S8).

### 2.12 Temporal response function (TRF)

We used the temporal response function (TRF) framework to relate brain activity to the stimulus. In order to estimate the overall representation of the stimulus in the neural data, we first adopted a decoding approach, in which the continuous stimulus could be reconstructed from multivariate neural data, either discrete or continuous. Under this framework, the stimulus is assumed to be the result of the convolution of neural data with a response function. TRF computations were performed using the spyEEG library (Guilleminot et al., 2026), with time window ranging from -0.5 to 0.5. Prior to model fitting, both stimulus and neural data were z-scored.

Because of the high dimensionality induced by the number of channels and lags, TRFs were estimated using a regularized deconvolution procedure rather than standard ridge regression. For each patient, the optimal regularization parameter (λ) was selected by nested cross-validation, testing 20 different values ranging from -3 to 3. Reconstruction accuracy was quantified using Pearson r between the reconstructed and original stimulus. For each signal representation (discrete, continuous and random continuous) and each binarization threshold, the number of channels and lags was held consistent, ensuring that all models had the same number of parameters and could be compared directly. Analysis were performed both at the subject level (many electrodes to one stimulus) and then at the single-electrode level, using an encoding framework, as to evaluate the contribution of individual channels.

### 2.13 Avalanches transition matrix (ATM)

We studied the propagation patterns of avalanches computing the ATM, from the recorded activity in a similar way as in Sorrentino, Seguin, et al. (2021). Following the estimation of the APr described above, we focused on single neuronal avalanches, defined as sequences of continuous activity (i.e., when at least two consecutive time steps of the APr are non-zero) starting after and ending before a zero-value bin of the APr. We first constructed an avalanche-specific ATM wherein the ATM(*i,j)* entry represents the probability that channel *j* is above threshold (i.e., it is 1 in the binarized data), at time *t+1* given that channel *i* was above threshold at time *t.* The participant- and condition-specific ATM_subject,condition_ is then built as the mean of the previously computed matrices across avalanches. A scheme of the ATM computation process is illustrated in Figure 5A. Note that the ATM is computed for each single avalanche lasting two or more time points. Then for a specific subject and condition, the full ATM is obtained by averaging across all avalanches recorded in that subject during that conditions. So in the end for each participant there is an averaged ATM for speech (averaged on the avalanches observed during speech listening), one averaged ATM for music and one averaged ATM for rest.

ATMs were estimated for each condition at the whole brain level, but also analyzed by separating auditory (Heschl’s gyrus, H) and non-auditory (noH) channels to estimate ATMs within (H–H, noH–noH) and between (H–noH; noH–H) them. Auditory channels corresponded to all the channels within the electrode that was traversing the Heschl’s gyrus, as defined visually by the neurosurgeon during the implantation procedure. The similarity of the ATMs between conditions was estimated as the absolute value of the Pearson’s correlation between the vectorized (excluding the main diagonal) ATMs. The stability of the distributions of the correlations was tested using a bootstrap approach. Statistical significance of the difference of Pearson’s correlation between pairs of conditions was evaluated via the Wilcoxon test.

### 2.14 Low fluctuations analysis

For the analysis performed on the lowest fluctuation, we firstly selected a threshold to ensure the same number of bins with a value of 1 as in the other analyses. The binarization process was performed similarly, with the key difference being that a bin was assigned a value of 1 if the absolute value of the z-score was below the threshold for at least one of the time steps corresponding to the bin. The results of this analysis are plotted alongside those from the high-amplitude bursts, using dotted lines in Figure 1B and Supplementary Figure S6.

### 2.15 Power law and branching ratio analysis

The study of the distribution of avalanche size and duration has been done by using the python toolbox *powerlaw* (Alstott et al., 2014), publicly available at the following link, https://github.com/jeffalstott/powerlaw. The comparison has been done by using this toolbox to compute the likelihood ratio between the power law fitting and the exponential. For what concern instead the branching ratio, σ, it has been computed as in Sorrentino, Seguin, et al. (2021), following the formula:

$$\sigma \;=\;e^{\frac{1}{N}\sum_{t'}\frac{APr\left(t+1\right)}{APr\left(t\right)}}$$

where the sum is done on all the time steps *t’* for which both APr(*t’*)>0 and APr(*t’* + 1)>0 and *N* is the total number of times steps *t’*.

## 3 Results

### 3.1 Neuronal avalanches support inter-subject correlations during speech and music

We first analyzed the timing of neuronal avalanches, defining their activity profile (APr) as the sum of the binarized data across channels (Fig. 1A), which measures the number of channels participating in a neuronal avalanche at each time-point. We computed the inter-subject correlation of the APr over time, for each condition (Fig. 1B; see Methods). We observed a significant inter-subject correlation between the APr of different participants during both speech and music listening, but not during rest (permutation tests: speech: mean correlation = 0.009, standard deviation (SD) = 0.014, p < 0.001; music: mean = 0.007, SD = 0.015, p < 0.001; rest: mean = 0.0007, SD = 0.014, p = 0.29; Fig. 1B). These inter-subject correlation results remained significant after excluding the 8-year-old participant (Supplementary Fig. S12) and were consistent across individual participants (Supplementary Fig. S13). The APr extracted from only the auditory cortex were significantly correlated across participants listening to the same acoustic stimulus, indicative of a stimulus-driven response (speech: mean = 0.032, SD = 0.021, p < 0.001; music: mean = 0.034, SD = 0.03, p < 0.001; rest: mean = 0.001, SD = 0.009, p = 0.062; Supplementary Fig. S7A). Moreover, when considering only the APr of a distributed network of non-auditory associative cortical regions, a significant inter-subject correlation was observed for speech, but not for music nor for rest (speech: mean = 0.003, SD = 0.013, p = 0.007; music: mean = 0.001, SD = 0.013, p = 0.09; rest: mean = 0.0001, SD = 0.013, p = 0.41; Supplementary Fig. S7B). In order to exclude the possibility that the inter-subject correlation was a byproduct of synchronous activations of spatially contiguous channels, we evaluated the inter-subject correlation for random picks of recording channels belonging to different anatomical regions (hence being spatially distant). Also in this case, a non-trivial correlation was observed during speech and music (but not rest) conditions (speech: mean = 0.0021, SD = 0.0006, p < 0.001; music: mean = 0.0017, SD = 0.0006, p = 0.003; rest: mean = 0.0003, SD = 0.0004, p = 0.261; reported mean and standard deviation are computed across iteration; Fig. 1C; see Supplementary Fig. S7C for assignment of channels to anatomical regions). Although the absolute correlation values are small, the large number of time samples included in the analysis (>100,000 per condition) provides high statistical power, allowing small but reliable effects to be estimated precisely and to reach statistical significance, as commonly observed in large-scale datasets (Cheng et al., 2024).

Our results highlight that speech and music processing are distributed processes, involving networks of brain regions activating together in response to external stimuli (Fedorenko & Thompson-Schill, 2014). To establish the role of high-amplitude burst of activity, we computed the intersubject correlation, when considering only the same amount of lowest fluctuations of the signal, dotted lines in Figure 1B. For each brain signal, we selected time points where the absolute value of the z-score was below a threshold, which was set to ensure the same number of time points as in the previous analysis. In this case, we observed non-significant correlation for all conditions (speech: mean = 0.0005, SD = 0.0058, p > 0.05; music: mean = -0.0002, SD = 0.0060, p > 0.05; rest: mean = -0.00001, SD = 0.0060, p > 0.05). This confirms the role of high-amplitude events of enhanced activity in shaping music and speech neural dynamics.

The binarization step requires fixing two main parameters: the threshold above which a channel is considered active, and a bin size that is used for temporally coarse-graining the data (see Methods). Thus, in order to check for the stability of our results we computed the inter-subject correlation for different values of threshold and bin size parameters. We observed that the thresholds at which the inter-subject correlation is maximal for both speech and music, and mostly different from rest, are above 95 percentile (Fig. 1D). This shows that focusing on rare salient events (i.e., using a high threshold when binarizing), while computationally more efficient, also selects features that map more closely on cognitive processes in the brain.

The binning process defines a temporal coarse-graining of our data, obtained by dividing each channel’s binary activity in time bins of size *m* and assigning 1 to bins when at least one activation was observed, and 0 if the channel was never active during the *m* time steps. The binning step allows us to look at the data with different time resolutions, and it is often used in the literature for example, to search for evidence of criticality in brain data (Fontenele et al., 2023), avoiding random noise effects in the scale-free distribution of neuronal avalanches size and duration. Here, we observe a peak in the inter-subject correlation for speech at bin size *m ≃ 100*, corresponding to ~1 second (Fig. 1E, green). For music, the inter-subject correlation reaches its maximal values at longer temporal scales around ~3 seconds (Fig. 1E, orange). This suggests that these two conditions may be characterized by different time scales of neuronal processing. As expected, the mean inter-subject correlation remains at baseline during rest, regardless of the bin size (Fig. 1E, blue). In the following analyses, we fixed *m=20 msec*, (and threshold = 99 percentile). This way, our results are not biased by the characteristic timescales of speech and music processing, while maintaining a high temporal resolution.

### 3.2 Neuronal avalanches are distributed during speech and music processing

We performed the topographic analysis of neuronal avalanches during speech, music, and rest, by analyzing the Activity Engagement (AE), a metric obtained by summing the binarized data over time (Fig. 1A), and quantifying the number of times each region is recruited by an avalanche event (a similar measure is also adopted in Bansal et al. (2021)). We hypothesized that the AE would differ across conditions. To test this, for each participant and for each channel i, we compared the AE(i) across pairs of conditions (i.e., speech versus rest, music versus rest, and speech versus music). For each participant and channel we compared AE across pairs of conditions, testing each channel against a null distribution (see Methods); channels surviving Bonferroni correction were labeled “significant-AE channels” (Supplementary Fig. S9A).

**Fig. 2.**
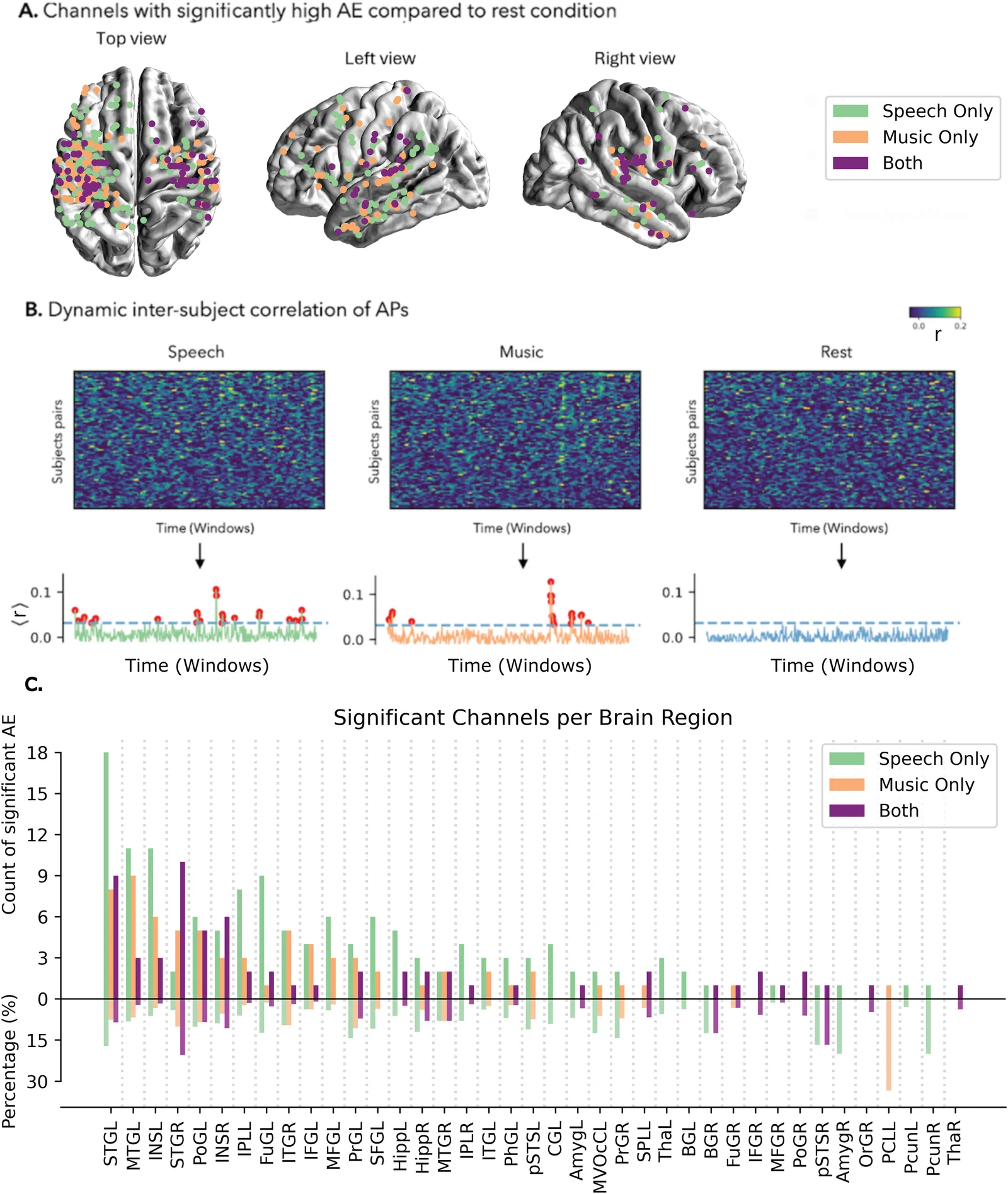
(A) Channels with significant activity engagement (AE, i.e., the sum over time of the binarized activations) compared to rest. Channels in green (/orange) mark significant AE during speech (/music) compared to rest, and channels in purple mark significant AE during both speech and music when independently compared to rest. (B) **(top)** Dynamic inter-subject correlation of APr plotted per time window (3 seconds) and for each pair of participants. **(bottom)** Evolution over time of the mean inter-subject correlation during speech (green), music (orange) and rest (blue). The red dots correspond to the windows of time in which the inter-subject correlation is higher than the maximum observed during rest (dashed blue line). Here, for plotting purposes the sliding step was 1 second, see Methods. (C) Spatial distribution of significant AE (AE significantly different during speech and music compared to rest). For each cortical region, the number of channels significant for speech and not for music is plotted in green, for music and not speech in orange and in violet for the ones significant for both speech and music. APr, Activity Profile; AE, Activity Engagement; ATM, Avalanche Transition Matrix; H, Heschl’s gyrus; noH, non-Heschl’s regions.

This analysis reveals that channels engaged during speech and music processing are spatially distributed and partially overlapping between conditions (Fig. 2A). While similar results have been observed with the more classical analysis of HFa amplitude (te Rietmolen et al., 2024), importantly, the analysis of activity engagement (AE) is complementary to the analysis of HFa, as these metrics are poorly correlated (Supplementary Fig. S10). Then, to gain more insights about which regions had significantly different AE during speech (or music) with respect to rest, we used the connectome atlas. We estimated the number of significant channels present in each of these regions. Most significant channels were located in the STG and MTG (Fig. 2C). Interestingly, the right STG was dominated by channels responding significantly to music only, while left STG was dominated by channels responding significantly to speech only. This confirms prior studies showing distinct hemispheric asymmetry in response to speech and music (Albouy et al., 2020; Zatorre et al., 2002).

### 3.3 Inter-subject correlation dynamics

To understand whether inter-subject correlations of APr are driven by specific coordinated events, we correlated the APr in a time-resolved way, using a sliding window approach with 3 seconds window size (see Methods). The mean inter-subject correlation value fluctuates over time (Fig. 2B), suggesting that there are specific moments in which neuronal avalanches of different participants listening to the same stimulus co-occur. In several time windows during speech and music listening, the inter-subject correlations indeed display high values, above the maximum observed during rest (red dots in Fig. 2B, bottom).

### 3.4 Neuronal avalanches encode speech and music dynamics

The results described above suggest that the binarized activity underlying neuronal avalanches is not arbitrary but is closely related to stimulus processing. Specifically, the presence of elevated inter-subject correlations, transient peaks in their temporal dynamics, and the spatially distributed pattern of channels with significant activity engagement (AE) all point toward a stimulus-driven origin of avalanche activity during speech and music listening. To directly test this hypothesis, we quantified the relationship between the binarized neural activity and the stimuli using temporal response function (TRF) modeling. Both speech and music were represented by their acoustic envelope alone, a low-level feature shared across stimulus categories and the standard input in TRF literature. We performed this analysis at two complementary levels. First, at a global level, we assessed whether whole-brain avalanche activity could be used to decode the stimulus. Second, at a local level, we used a single-channel encoding approach to evaluate the influence of binarization on the contribution of individual recording sites.

At the global level, stimulus decoding was performed using a multivariate TRF approach, in which the stimulus was reconstructed from the combined activity across channels via linear regression with a learned response filter. To disentangle the effects of binarization and data sparsification, we compared three signal representations. The *discrete* representation corresponds to the neuronal avalanche signal, in which data points above a given threshold are retained as binary events (value = 1) and all others are set to zero. The *continuous* representation applies the same threshold but preserves the original signal amplitude and sign for retained time points. Finally, the *random continuous* representation retains the same number of time points as the continuous case, but selects them randomly, allowing us to isolate the contribution of temporal structure from simple sparsity.

**Fig. 3.**
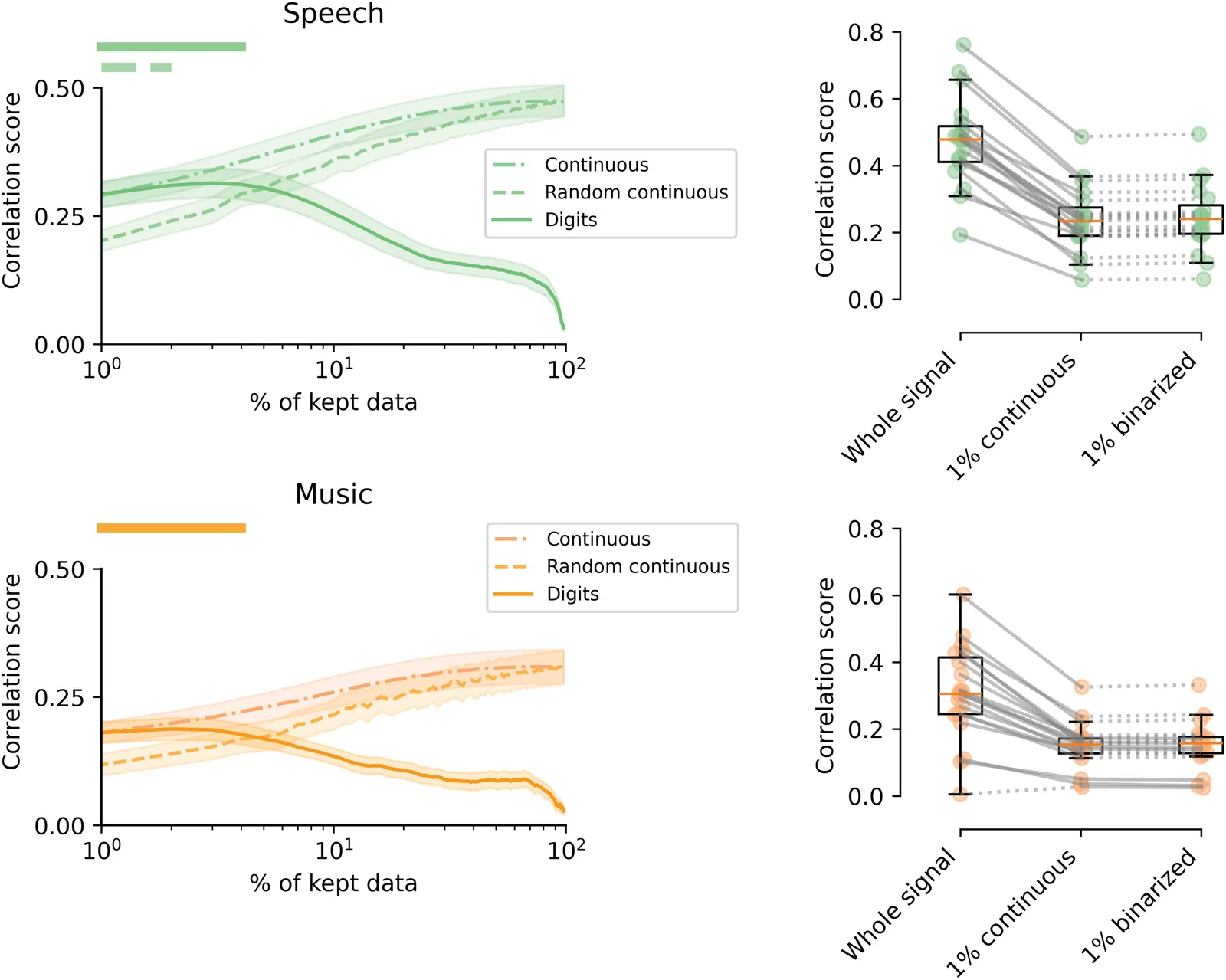
**(Left column)** Correlation between the predicted and actual stimulus as a function of the percentage of retained data for speech (green) and music (orange). Temporal Response Function (TRF) analysis to decode the stimulus envelope dynamics are computed for three neural signal representations: continuous signals in which only the top n percentile of values is retained and the rest set to zero (dash–dotted); randomized continuous signals in which the same number of time points corresponding to the n percentile is randomly selected and all others set to zero (dashed); and binarized signals in which activity is thresholded at the n percentile and converted to binary values, as in the avalanche analysis (“digits”, solid). Shaded areas denote the standard error across subjects. The continuous and dotted lines above the plots index interval of thresholds in which the distribution of correlations across subjects was significantly higher for the binned signal with respect to the continuous (dotted) and random continuous (continuous). These significance values were obtained performing t-test statistics and p-values (p minor 0.05 was considered significant after correction for multiple comparison—for the number of different thresholds tested). **(Right column)** correlations are shown at the single-subject level. We compare TRFs computed from the HFa signal with those computed from a sparse representation retaining the top 1% of time points while preserving their original sign and amplitude (values below threshold set to zero, without binarization). Grey dashed and continuous lines connect the values of the same participants across the three distributions. When the scatter point at the left end of the line is lower than the one at the right end of the line, the line is dotted, otherwise it is continuous. TRF, temporal response function; HFa, high-frequency activity.

Because a key parameter of the avalanche framework is the threshold used to binarize the signal, we systematically explored a wide range of thresholds, from the 0th to the 99th percentile. Across thresholds, we found that stimulus–response correlations remained high even when up to 99% of data points were discarded. Notably, for thresholds below the 95th percentile, the discrete (binarized) representation consistently outperformed the random continuous control, indicating that meaningful information is preserved in the timing of threshold-crossing events. At the 99th percentile, particularly for speech, the discrete representation yielded higher decoding performance than the continuous representation across all subjects. This suggests that, for the most extreme activity bursts, binarization preserves stimulus-related information more effectively than retaining the original signal amplitude, for which sign and magnitude appear to contribute mainly to noise (Fig. 3).

**Fig. 4.**
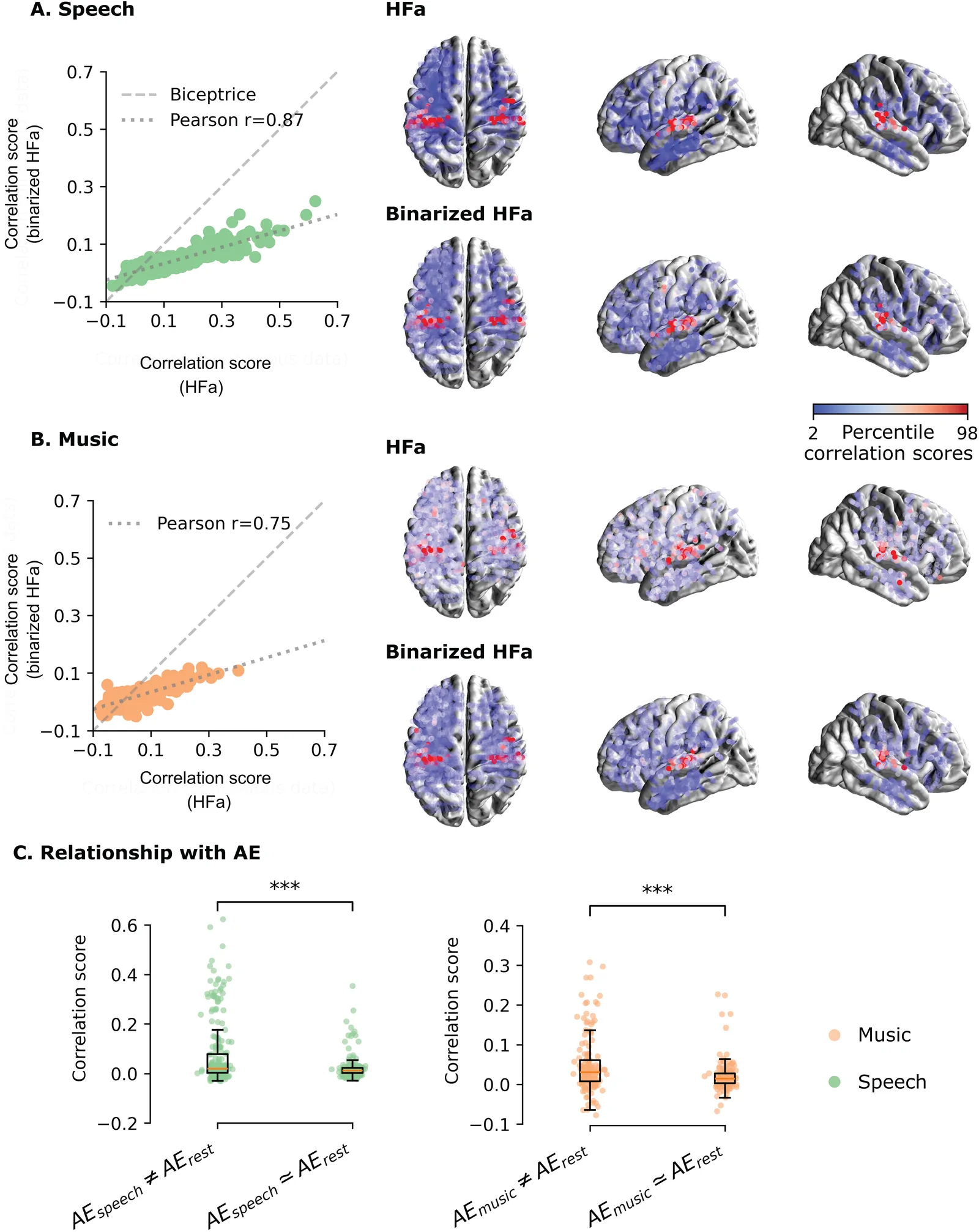
Brain topographies for speech (A) and music (B). Colors indicate cross-validated encoding performance (5-fold), quantified as Pearson’s correlation between predicted and actual brain activity using single-channels TRFs. Results are shown for continuous HFa (top) and binarized HFa (bottom; thresholded at the 99th percentile, as in the avalanche analysis). Scatter plots (left) compare encoding for continuous and binarized HFa, showing highly similar spatial patterns and performance despite retaining only the top 1% of time points. (C) Encoding performance for channels with significantly different activity engagement (AE) during speech or music relative to rest. Channels with significantly different AE during speech/music show significantly higher correlation scores than channels with AE not significantly different from rest; for both speech (green) and music (orange). This analysis directly links AE to stimulus encoding. TRF, temporal response function; AE, activity engagement. ***p < 0.001.

We next extended this analysis to the single-channel level. Here, TRF analysis was performed independently for each channel, allowing us to relate local stimulus encoding to activity engagement. Notably, also when discarding the 99% of data points, the correlation between the topographies of correlation scores found for HFa and binarized activity were significant (Pearson correlation = 0.87, p<0.001 for speech and Pearson correlation coefficient (r) = 0.75, p<0.001 for music; Fig. 4A, B). Channels that exhibited significantly different AE in previous analyses also showed the highest stimulus decoding correlations, with correlation score distributions that were significantly elevated compared to the remaining channels (p-values <0.001, for both speech and music; Fig. 4C). This relationship is not trivial, as the AE measure itself is computed without any reference to the stimulus and relies on a simple, computationally efficient binarization of the signal. Nonetheless, the convergence between AE and TRF decoding results indicates that avalanche-based measures capture stimulus-relevant neural dynamics and may provide a compact and informative representation of large-scale neural processing during speech and music perception.

### 3.5 Neuronal avalanche propagation pathways change between conditions

**Fig. 5.**
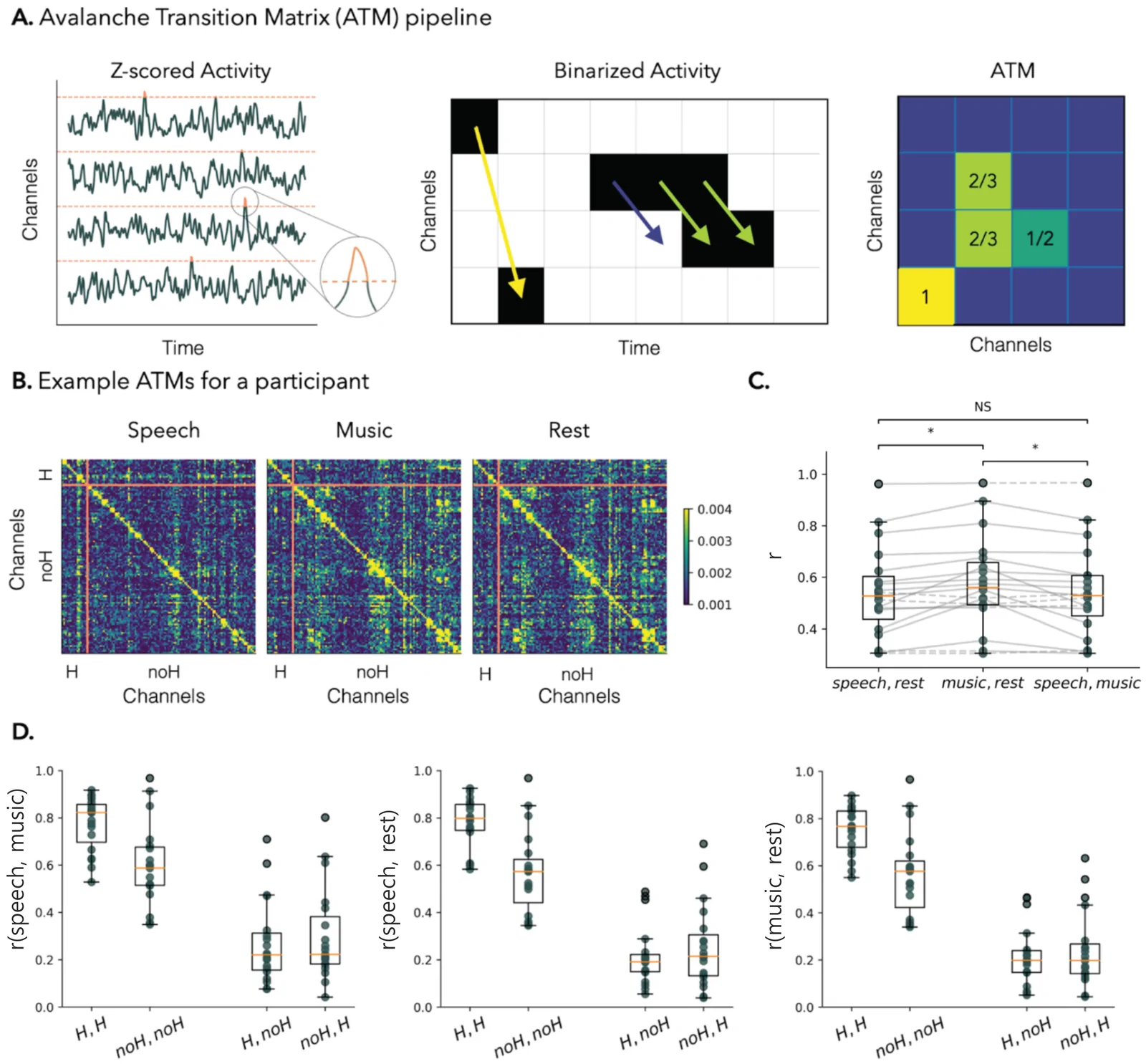
(A) Avalanche transition matrices (ATMs) pipeline. **(left)** Example of binarized activity extracted from the HFa. **(middle)** For each pair of channels i,j of a participant, the transition of activity between channel i at time t and channel j at time t+1 is estimated. **(right)** Each element in position i,j of the ATM represents the probability that channel j records a salient activation at time t+1 given that channel i recorded a salient activation at time t. (B) ATMs of one participant for the three conditions. The orange lines separate the four subsets of ATMs’ elements investigated (H–H, noH–noH, noH–H, H–noH). (C) Distribution of Pearson’s correlation values (corr) across participants, computed between ATMs of pairs of conditions. Solid/dashed gray lines: participants showing the group-average/reversed effect (Wilcoxon test: *p < 0.01; NS non-significant). (D) Distribution of correlation values across participants, computed between ATMs of pairs of conditions. ATMs were estimated within (H–H, noH–noH) and between (H–noH; noH–H) areas. The boxplots in panels C-D represent the median and the interquartile range. H: Auditory (Heschl’s gyrus); no-H: non-auditory channels. ATM, Avalanche Transition Matrix; H, Heschl’s gyrus; noH, non-Heschl’s regions; TRF, temporal response function; Corr, Pearson Correlation.

In order to further investigate the speech, music, and rest conditions, we analyzed the spatiotemporal propagation of neuronal avalanches through the brain. We computed the avalanche transition matrices (ATMs) (Duma et al., 2023; Sorrentino, Seguin, et al., 2021), which measure the probability that the activation of channel i results in the activation of channel j at the successive time step (Fig. 5A; see Methods). Hence, for each participant, the ATMs estimate the characteristic pathways through which neuronal avalanches propagate. We computed the ATMs for each condition (example participant in Fig. 5B) and compared them by correlating their respective (vectorized) ATMs (Fig. 5C). The ATMs of rest (r) and music (m) are significantly more correlated than the ATMs of speech (s) and rest, or speech and music (Wilcoxon test: p = 0.003 for (s,r) versus (r,m); p = 0.86 for (s,r) versus (s,m); p = 0.006 for (r,m) versus (s,m)). This suggests that avalanche propagation during music listening and rest is more similar than during speech listening.

To disentangle the contribution of local auditory (intra-areal) and distributed (inter-areal) processes we separated auditory (Heschl’s gyrus, H, recorded with a total of 345 channels over the population of the present study) and non-auditory (noH) channels, and estimated the ATMs within (H–H, noH–noH) and between (H–noH; noH–H) them. Note that, as ATMs estimate directional transitions, the H–noH ATM refers to avalanches projecting from auditory to non-auditory regions, while the noH–H ATM to neuronal avalanches incoming to the auditory cortex. Next, we analyzed the correlations between the ATMs by focusing on the H–H, noH–noH, H–noH, and noH–H blocks separately. As for the previous part, these correlation values are computed across conditions, hence a correlation between speech and music for a specific subject measures how much its ATM during speech and music listening was similar. In this last part of analysis, we computed this correlation separately considering only the edges from H to H, from H to noH, noH to H, and noH to noH in order to measure how much a group of edges of interest (H to H, noH to H, H to noH, or noH to noH) is similar across conditions. In this way, we obtained a correlation value per subject, H/noH edges groups and pairs of conditions, and we observed that for all the subjects H–H and noH–noH groups were always more correlated across speech and music conditions with respect to the other groups of edges.

The correlations between the ATMs in H–H and noH–noH are higher than those considering H–noH and noH–H blocks, consistently across all pairs of conditions (Fig. 5D; comparing in each panel the first two distributions to the last two; paired non-parametric Wilcoxon signed-ranks tests: all p < 0.001; all z-statistic >3.7). This indicates that the functional connections that are changing the most between conditions are the ones between auditory and non-auditory regions (either projecting from or going into H-channels), while the connections between channels localized both within (H–H) or outside (noH–noH) the auditory cortex are more stable across conditions. Moreover, the high correlation values of ATMs in H–H across all pairs of conditions suggest that the transition matrix does not vary much within the auditory cortex across conditions. Correlations in H–H are higher than those in noH–noH; all p < 0.002; all z-statistic >3.1.

Finally, the correlations between the ATMs in H–noH and noH–H are significantly different only when comparing speech and music (Fig. 5D, left panel; p = 0.036), but they are not statistically different when comparing speech to rest or music to rest (Fig. 5D, central and right panels; p > 0.08). In all cases, the ATMs correlations in the H–noH and noH–H blocks are rather low (Pearson’s correlation ~0.2). These results indicate that the (feedforward and feedback) propagation of neuronal avalanches between auditory and associative regions best indexes the specificity of cognitive processes induced by different conditions.

## 4 Discussion

Naturalistic stimuli such as movies, virtual reality, speech, or music listening create an ecologically-relevant set up to study the emergence of neuronal processes and the mechanisms supporting brain functioning. In the present work, we investigated two major auditory cognitive domains, namely speech and music processing. As both domains are universally and uniquely human, scientists, since Rousseau and Darwin, have debated whether speech and music share common or different origins. In neuroscience, this debate can be addressed by assessing how these processes are dynamically mapped into brain activities.

Brain mapping requires processing and analyzing brain signals, for which there exists no unique approach. In fact, as a complex system, the brain can be effectively studied according to different perspectives. A canonical perspective to interpret brain data is in terms of brain rhythms or oscillations (Buzsáki, 2006; Buzsáki & Moser, 2013). Temporal analysis and spectral decomposition of “analog” signals allow isolating frequencies of interest with a putative role in a specific brain function (Fries, 2015). A complementary perspective to study information processing in the brain is offered by the analysis of high-amplitude events, which involve distributed brain regions (van Ede et al., 2018). In fact, from the early cybernetic interpretation of the brain as a “digital” machine (Heims, 1991) to the more recent applications of information theory tools (Timme & Lapish, 2018), it was shown that a great deal of information can be extracted by binarizing the signals. In this work, we demonstrated this principle by analyzing an sEEG dataset during naturalistic acoustic stimuli preserving only a small percentage of data points corresponding to neuronal avalanches. This approach effectively retrieved relevant information about brain processing during speech and music listening, including intersubject correlations (Fig. 1), and characteristic topographies of activations (Figs. 2 and 3).

Binarizing signals presents a compelling computational advantage, demanding significantly lower computational resources compared to the analysis of temporal and spectral signal properties. Notably, the binarization process employed in this work retains only approximately 1% of the data points. Despite this substantial reduction in the volume of data considered, the predictive power of binarized activity remains robust. In fact, decreasing the amount of data can enhance the quality of our results, as illustrated in Figure 1D, where higher binarization threshold led to stronger inter-subject correlations. This underscores the efficiency and effectiveness of signal binarization in optimizing computational resources without compromising analytical insights.

Even though in each participant the channels were placed in different locations, we found significant inter-subject correlations of the APr, suggesting that the processing of speech and music in the human brain does not involve only some specialized regions, but rather a degenerate computation (Bernard, 2023; Edelman & Gally, 2001) involving different brain areas sharing similar temporal dynamics. This result is further strengthened by our analysis performed on a sparse channel selection, where using only one channel per anatomical region preserved significant inter-subject correlations (Fig. 1C).

Our experiment, conducted in ecologically valid conditions, allowed participants to naturally process speech and music across various levels, from phonemes to sentences, and from single notes to musical phrases. During speech listening, we observed significant inter-subject correlation also after excluding the channels in auditory regions (Supplementary Fig. S7). This might reflect integrative “high-level” stimulus-driven cognitive processes during speech listening. While this was not observed during music, we expect that a temporal coarse graining would reveal a similar feature also during music listening, in virtue of the better performances shown by large binning values during music listening (Fig. 1E). Alternatively, the divergence could stem from individualized processing of music phrases as compared to speech sentences, where the linguistic/semantic processing during storytelling temporally aligns across subjects, while personal experiences differ across subjects listening to music.

These results confirm previous work that describes speech and music processing as distributed across the whole-brain (Price, 2000; Warren, 2008). However, it is still unclear whether these processes are related to language/music-specific regions or if, rather, they rely on shared networks (Peretz et al., 2015). Previous work on the same dataset analyzed in this manuscript showed that, in terms of both HFa and other frequency bands, the spatial specializations for speech and music are, in fact, alike: they do not recruit selective cortical regions but mostly rely on shared resources (te Rietmolen et al., 2024). Our analysis of neuronal avalanches topographies (using the rest condition as a baseline) partially confirms these results, as several channels with significant-AE were shared between speech and music processing (Fig. 2A, C, purple channels). This was particularly evident in auditory areas, though not exclusively. Away from these areas, music and speech were characterized by significant-AE in different subnetworks (Fig. 2A). This confirms that these two conditions rely on partially overlapping whole brain networks hinging across different brain areas. A direct comparison of the speech and music conditions confirms the differences between speech and music processing (Supplementary Fig. S8D), but only in a small percentage (less than 10%) of the channels (Supplementary Table S1; te Rietmolen et al., 2024).

Moreover, the observed spatial topography of activity engagement in avalanches dynamics is broadly consistent with previous work on the cortical processing of speech and music. The prominence of bilateral auditory regions, particularly the STG, aligns with extensive evidence implicating these areas in the analysis of complex acoustic signals (Albouy et al., 2020; Leaver & Rauschecker, 2010; Norman-Haignere et al., 2015; Peretz et al., 2015; Sankaran et al., 2024; Tang et al., 2023; Zatorre et al., 2002; Zuk et al., 2020). The relative dominance of speech-related effects in the left STG and music-related effects in the right STG mirrors well-established hemispheric asymmetries reported for speech and melodic processing (Albouy et al., 2020; Zatorre et al., 2002). Importantly, the involvement of associative temporal and frontal regions suggests that avalanche dynamics reflects large-scale network coordination during naturalistic listening, extending beyond early auditory processing. Together, these findings support the view that speech and music engage partially overlapping but asymmetrically distributed cortical networks, whose coordinated dynamics can be captured through the investigation of neuronal avalanches topography.

In addition to the spatial organization of high-amplitude bursts, our analyses directly link these events to the acoustic structure of the stimuli. Temporal response function (TRF) analysis revealed robust encoding and decoding performance based on burst activity. At the population level, decoding analyses showed high correlations between real and predicted stimulus features (positive correlation score for all the 19 participants and mean Pearson r = 0.24), while at the single-channel level, encoding analyses demonstrated reliable prediction of neural responses from the stimulus (positive correlation score for all the 19 participants and mean Pearson r = 0.15). Previous work has shown that discretizing the external stimulus can improve TRF-based encoding (Oganian & Chang, 2019). Here, we extend this logic to the neural domain, demonstrating that discretizing brain activity, by selecting high-amplitude bursts, also yields robust encoding and decoding results.

Despite binarization discarding 99% of the original continuous high-frequency activity (HFa) signal, approximately 50% of the encoding and decoding performance was preserved (mean Pearson r = 0.49 for continuous HFa vs mean Pearson r = 0.24 for binarized HFa, during speech; mean Pearson r = 0.3 for continuous HFa vs mean Pearson r = 0.15 for binarized HFa, during music listening). Importantly, the spatial topography of stimulus encoding was largely maintained after binarization (spatial Pearson r = 0.75 for music; spatial Pearson r = 0.87 for speech). indicating that the functional organization of auditory processing is conserved in this sparse representation. Strikingly, restricting the signal to the top 1% of activations in binary form resulted in better decoding performance than retaining the same top 1% as continuous-valued data. Together, these findings indicate that high-amplitude bursts of high-gamma activity provide an efficient and compact representation of stimulus-related information. Rather than reflecting mere high-amplitude epiphenomena, these discrete network events preserve behaviorally relevant information about ongoing speech and music while dramatically reducing data dimensionality.

Finally, examining the propagation of neuronal avalanches (Fig. 5), we discovered that connections between auditory and non-auditory regions exhibit the least correlation across different conditions. This implies that the most dynamic changes between rest, speech, and music occur along the feedforward and feedback pathways. Additionally, functional connections outside the auditory regions demonstrate significantly lower correlation across conditions compared to those within. This shows that speech and music listening robustly modulate the information flow among frontal and temporal cortical regions, and less so in the auditory cortex itself.

When interpreting these results, it is important to consider the different degrees of spatial heterogeneity across recording sites within Heschl’s gyrus (H) and outside of it (noH). Channels outside Heschl’s gyrus span multiple cortical regions and are therefore more heterogeneous than channels within Heschl’s gyrus. In this context, our analysis focuses on correlations of avalanche transition matrix (ATM) values across different channel groupings (H–H, H–noH, noH–H, and noH–noH). Among these groupings, the noH–noH category is expected to exhibit the highest spatial heterogeneity, followed by cross-group interactions (H–noH and noH–H), with H–H being the most homogeneous.

Importantly, the observed results cannot be trivially explained by these heterogeneity differences. Specifically, the strongest differences emerge between within-group (H–H and noH–noH) and cross-group (H–noH and noH–H) interactions. This pattern runs opposite to what would be expected if spatial heterogeneity alone were driving the effects, as the most heterogeneous category (noH–noH) does not simply show reduced or degraded correlations relative to cross-group interactions. This dissociation suggests that the observed propagation patterns reflect genuine differences in stimulus-related dynamics rather than being a trivial consequence of anatomical heterogeneity. A more fine-grained spatial characterization of ATM propagation patterns would certainly be desirable. Nonetheless, this is challenging because ATM is defined on pairs of channels (rather than single channels) and brain coverage is highly heterogeneous across participants. Specifically, almost no pairs of regions in the Connectome brain atlas are sampled consistently across the cohort. Consequently, our spatial characterization was restricted to comparing H versus noH channels.

In this work, we focused on (the binarization of) HFa signals, which are generally used to provide insights into the neural bases of cognition (Lachaux et al., 2012). In general, the analysis of binarized signals offers a complementary view of brain data, which is only weakly related to more diffuse measures of brain oscillations (e.g., signal power; Supplementary Fig. S10). Typically, above-threshold activities are interpreted as higher-order dynamical properties. In Supplementary Figure S10, we show that AE and HFa power, computed as the total power over the recording for each channel, are not correlated with each other. This indicates that the two measures capture distinct aspects of the neural signal or at least that one is not trivially explaining the other. Nevertheless, both AE and HFa are related to stimulus envelope (Fig. 3) and are modulated by speech and music listening (Fig. 2). In this sense, these complementary analyses provide two different perspectives on the same neural activity, each reflecting stimulus-related processing, while not being trivially explained by one another.

In Supplementary Figure S11, we further show that the APr and AE measures introduced in this work, and obtained from the binarized activity, can be associated with the characteristic decay time of signals’ autocorrelation, a measure that in the “analog” signal processing framework has been used to measure the presence of characteristic timescales in brain activity (Fulcher et al., 2019; Gao et al., 2020; Golesorkhi et al., 2022; Müller & Meisel, 2023; Murray et al., 2014; Northoff et al., 2021; Sorrentino et al., 2023). In this context, the “digital” framework of the APr and AE could offer a time-resolved and computationally advantageous proxy of timescales dynamics. Moreover, the binarization process also allows us to probe the temporal scale of the signal from a complementary perspective. Specifically, we applied temporal coarse-graining by grouping consecutive time bins and assigning a value of 1 if at least one bin within the group was active. We repeated this procedure across a range of temporal bin sizes and found that the intersubject correlation peaked at longer temporal scales for music than for speech (Fig. 1E). This difference is consistent with the temporal modulation spectra of the stimuli, which showed a dominant peak around 2 Hz for music and around 4 Hz for speech (Supplementary Fig. S5; Ding et al., 2017), suggesting that the optimal temporal scale of shared high-amplitude events partly reflects bottom-up acoustic structure. However, this effect is unlikely to be purely acoustic. Naturalistic speech and music also impose distinct integration demands: speech comprehension requires the accumulation of phonemic, lexical, semantic, and narrative information over time, whereas music listening may involve the integration of rhythmic, melodic, and harmonic regularities over comparatively longer windows (Farbood et al., 2015; Lerner et al., 2011). Thus, the observed difference possibly arises from an interaction between stimulus-driven modulation statistics and top-down integration windows engaged by each stimulus class. Future work using stimuli in which acoustic structure and higher-level interpretability are independently manipulated could help dissociate these contributions more directly.

Besides computational efficiency, binarization allows the deployment of a series of conceptual tools such as information theory (Timme & Lapish, 2018), the study of network dynamics (Zhou et al., 2021), as well as paradigms such as brain criticality (Cocchi et al., 2017; Taylor et al., 2013). In relation to the latter, we highlight that, in standard neuronal avalanches analyses aimed at testing the presence of a critical state in the brain, the bin size value chosen for the data binarization plays an important role. In fact, the tuning of the bin size value is important to guarantee the observation of power-law distributed neuronal avalanches (Fontenele et al., 2023). Beyond the study of criticality, the bin size defines the time resolution by which we look at the data. Analyzing the intersubject correlations for different bin size values (Fig. 1E), we showed that speech and music processing are characterized by different timescales, with optimal performances for different bin size values. This suggests that, depending on the problem at hand, a convenient definition of the bin size can also be determined by functional performances, rather than by the search for any particular statistics of neuronal avalanches.

Among the limitations of our study, we must acknowledge the inability to conduct a more detailed analysis of criticality parameters of brain activity due to the limited recording duration and spatial sampling. Because Heschl’s gyrus was the only cortical region systematically recorded across all participants, as it was an inclusion criterion for the study, our spatially resolved analyses were necessarily restricted to this region. Additionally, although no seizures were recorded during the sessions, this does not rule out completely the presence of interictal events, which could potentially influence the topography presented in Figure 2, though they are unlikely to affect the observed temporal synchrony of the avalanches. Future work could explore avalanches across different frequency bands and examine the relationship between observed avalanches and the acoustic and semantic features of the naturalistic stimuli. Also, future work might include a deeper investigation of the spatial properties of ATMs to test whether the feedforward and feedback propagation patterns observed here can be mapped with finer regional specificity across the auditory cortical hierarchy and associated fronto-temporal networks. The choice of focusing on the temporal envelope of speech and music stimuli was motivated by evidence showing that it carries behaviorally relevant rhythmic information and is neurally tracked across both domains, providing a temporal scaffold for online auditory processing (Poeppel & Assaneo, 2020; te Rietmolen et al., 2024). However, next steps of analysis can focus on relating neuronal avalanches to different stimulus features, extending the current analysis focusing only on the stimulus temporal envelope. Finally, we acknowledge several methodological directions along which our analysis could be extended, including the direct investigation of information processing using recently developed information-theoretic approaches grounded in recent theoretical advances, as well as a focus on higher-order interactions to further unravel the distributed nature of speech and music processing (Combrisson et al., 2025; Neri, Brovelli, et al., 2025; Neri et al., 2024; Santoro et al., 2025).

## 5 Conclusion

In conclusion, our study provides compelling evidence that neuronal avalanches offer an effective means to characterize neuronal processing in naturalistic conditions. This provides a valuable complement to oscillation-based frameworks, thereby enriching the ongoing discourse on the neuronal underpinnings of speech and music processing.

## Supplementary Material

Supplementary Material

## Data Availability

The sEEG data used for the analysis in this study are available from the authors upon reasonable request. The code is available in the public repository, https://github.com/Mattehub/sorciere.
