# Supplementary material for: The role of high-amplitude bursts of high-gamma activity in naturalistic speech and music listening
Source: Imaging Neurosci (Camb). 2026 Aug 27;4:IMAG.a.1348. doi: 10.1162/IMAG.a.1348 (PMC13522956; doi:10.1162/IMAG.a.1348)
Supplement: Supplementary file 1 [file IMAG.a.1348_supp.pdf]

# Supplementary Material of the article: The Role of High-Amplitude Bursts of High-Gamma Activity in Naturalistic Speech and Music Listening

## Neuronal avalanches features.

Neuronal avalanches have been widely studied in the literature as a hallmark of criticality in brain dynamics. Although the present work did not specifically aim to address questions related to criticality, we nonetheless performed the standard analyses commonly used to assess whether brain activity operates near a critical regime, to support the use of the term neuronal avalanches. Specifically, we computed the size and duration of each neuronal avalanche and quantified the statistical properties of their distributions. Avalanche size was defined as the total number of regions active during an avalanche, whereas avalanche duration corresponded to the number of consecutive time points over which the avalanche persisted (Figure S1). Across all participants, the distributions of avalanche sizes and durations exhibited power-law–like behavior (Figure S2). In all three experimental conditions, the empirical data were better described by a power-law fit than by an exponential model. Another parameter employed in the investigation of criticality in the brain, is the branching ratio, that we computed as in previous literature (40). The branching ratios were consistently close to 1 across conditions, indicating dynamics near criticality, that it is consistent with prior studies (on rest). Speech exhibited a slightly higher branching ratio compared with music and rest, potentially suggesting a modest shift toward more critical-like dynamics; however, these differences were small, despite reaching statistical significance.

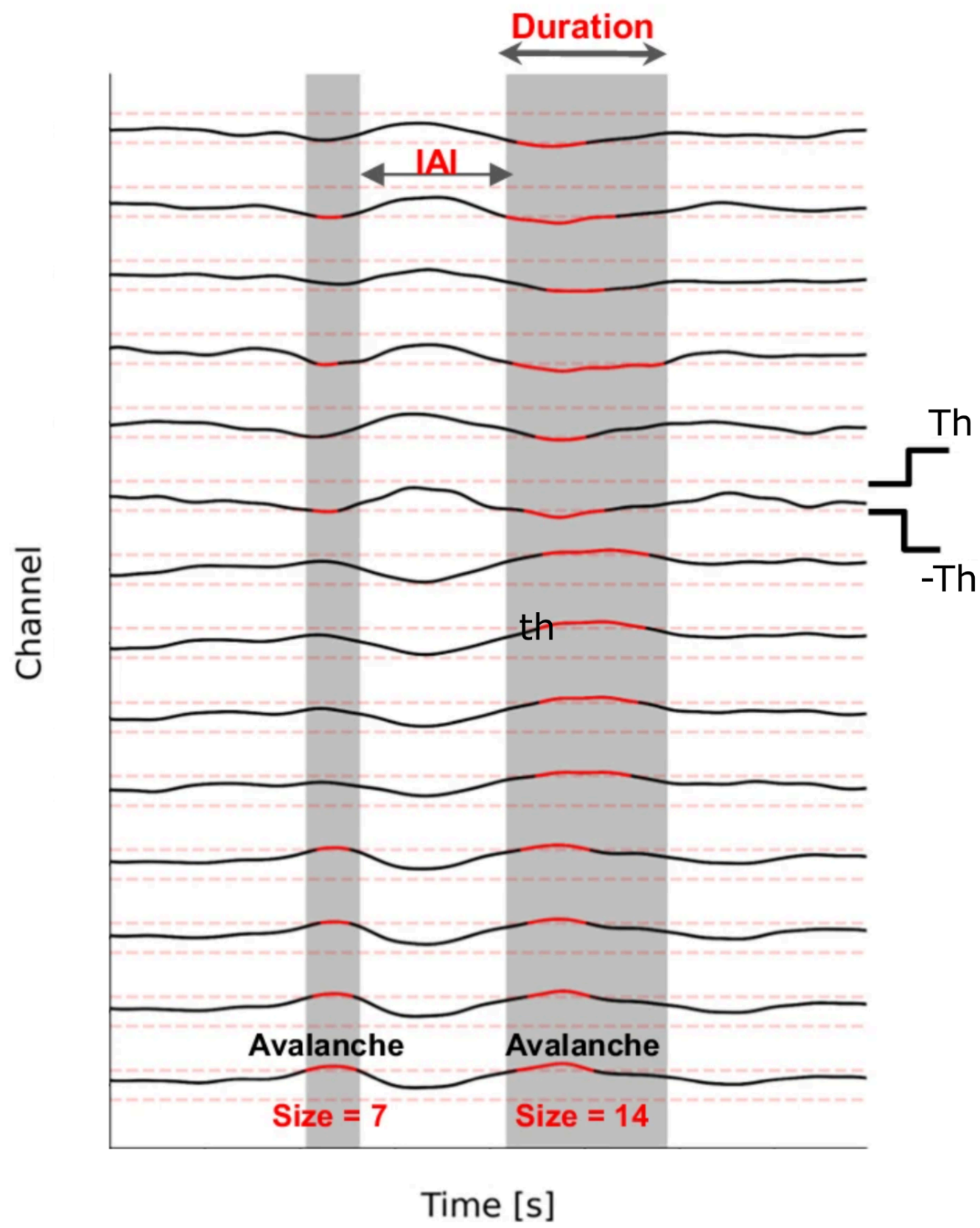


***Figure S1.*** *Multichannel time series are shown across recording channels. Activity exceeding a predefined threshold (±Th) is marked in red. An avalanche is defined as a sequence of consecutive time bins containing at least one suprathreshold event, bounded by silent bins. Avalanche* ***duration*** *corresponds to the number of consecutive active bins, while avalanche* ***size*** *is defined as the total number of suprathreshold events (or active channels) occurring during the avalanche.*

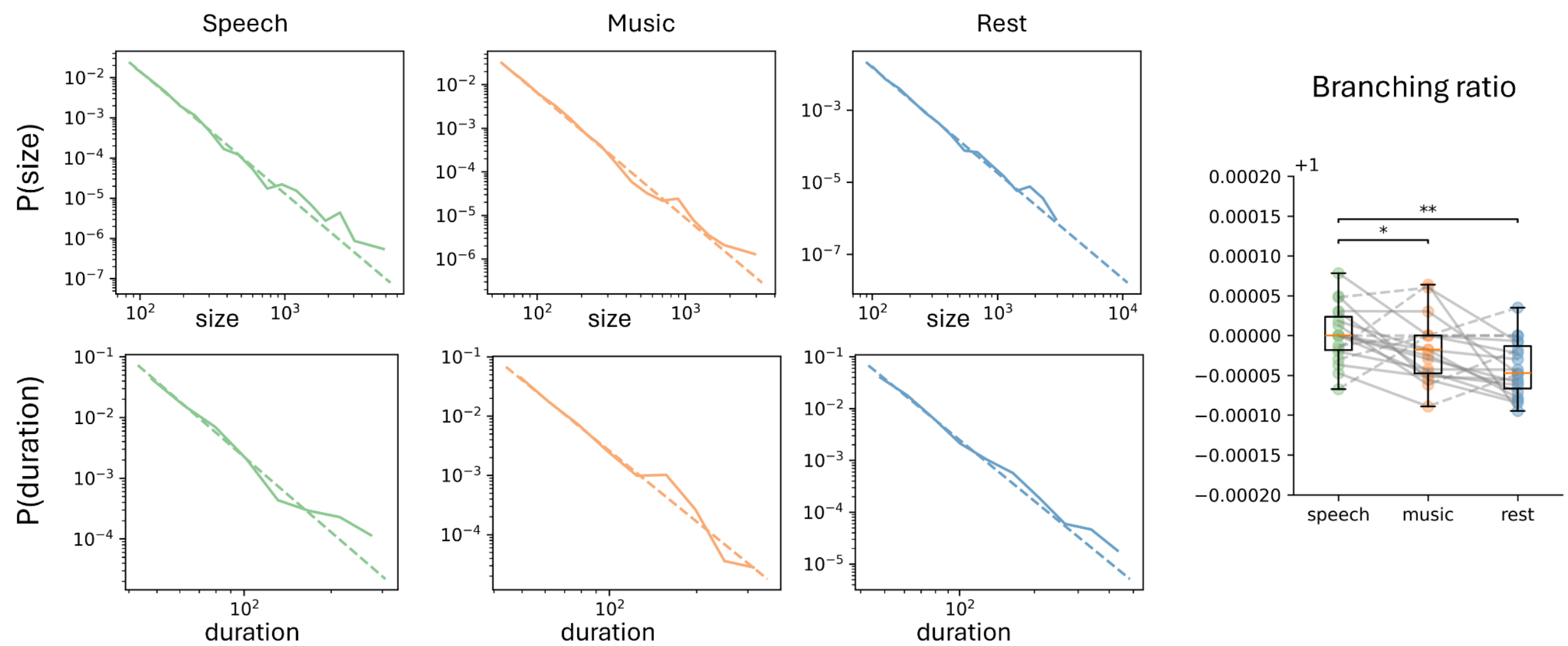


***Figure S2.*** *The distribution of avalanche sizes and durations is plotted for all avalanches observed across subjects. The dotted line represents the power-law fit, while the solid lines depict the actual data distributions. Avalanche size corresponds to the number of active bins during the avalanche, and duration refers to the total length of an avalanche, measured in bins (1 bin = 20 ms, see methods). The distributions more closely matched a power-law than an exponential model (avalanche duration: speech, R = 43, p = 0.00005; music, R = 34, p = 0.0001; rest, R = 52, p =0.0002. avalanche size: speech, R = 463, p < 10-8; music, R = 727, p < 10-14; rest, R = 403, p < 10-5). In the right panel, the branching ratio is shown. Colors are consistent with the rest of the manuscript: blue for rest, orange for music, and green for speech. During speech, the branching ratio was slightly higher than in music and rest, potentially indicating a better tuning to criticality, though the differences were minimal (average branching ratio: speech: 1.000001; music: 0.999982; rest: 0.999959; Wilcoxon signed-rank test: speech vs music, statistic = 32, p =0.035; music vs rest, statistic = 24, p =0.07; speech vs rest, statistic = 44, p =0.002).*

## Brain topographies

First of all, we systematically show the implantation of our cohort of participants. Reporting also the number of channels per participants as well as the number of regions recorded per participant (Fig.S3-4). As it is possible to observe our sample is strategically biased towards language and music areas, in particular the STG and INS. For all these analysis we used the brain connectome atlas, since it provided a good parcellation of brain atlasses, also with respect to previous literature.

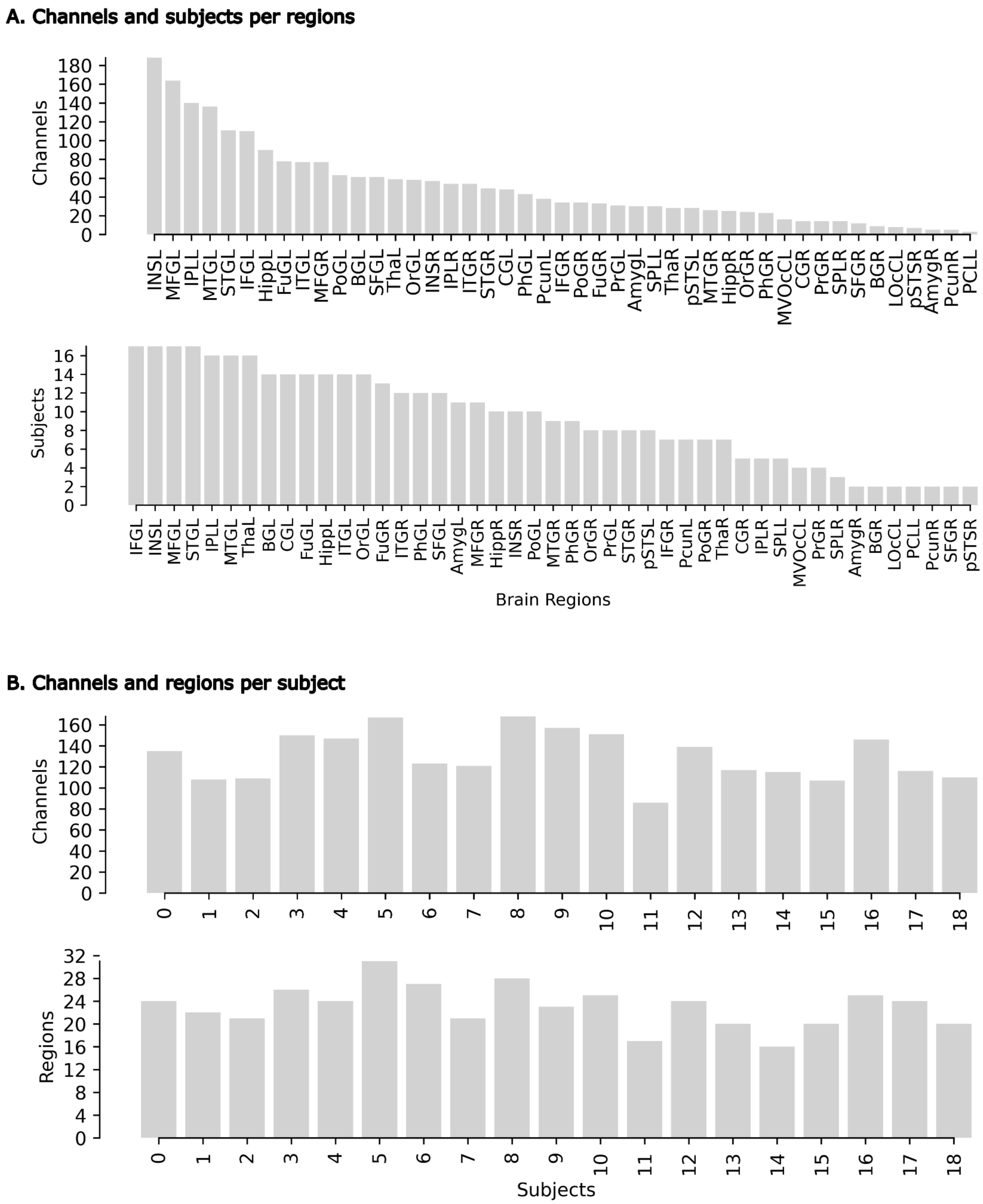


***Figure S3. Panel A**: Number of recording channels per brain region (top) and number of subjects in which each region was sampled (bottom), ordered in descending order. This illustrates the heterogeneous spatial coverage across regions, with frontal and temporal areas being most frequently recorded. **Panel B**: Number of recording channels (top) and number of sampled regions (bottom) for each subject, showing variability in electrode coverage across participants.*

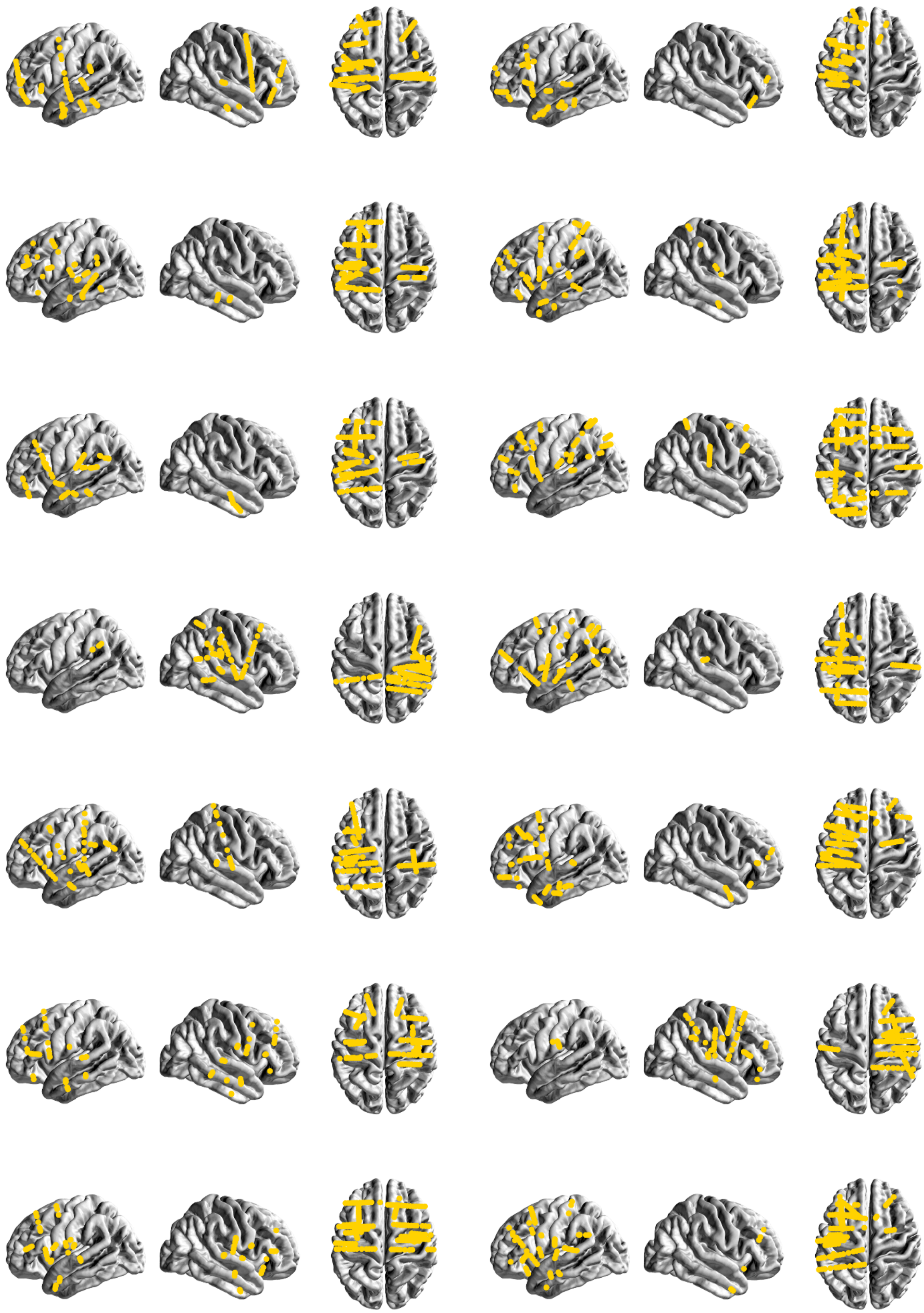

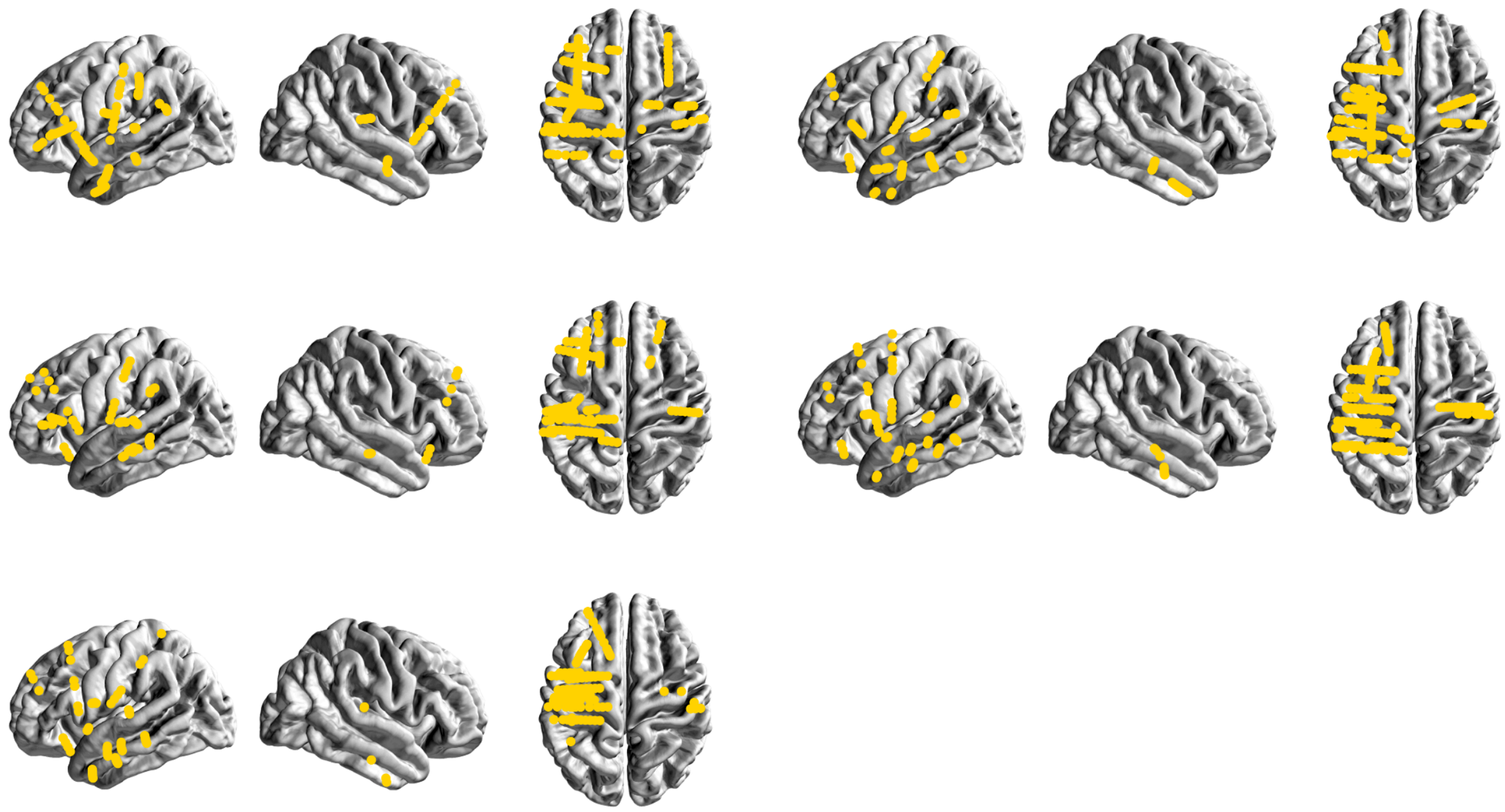

***Figure S4.*** *Anatomical localization of the stereotactic EEG (sEEG) electrodes for each patient (N=19, n channels =* 24*).*

## *Stimuli acoustic properties*

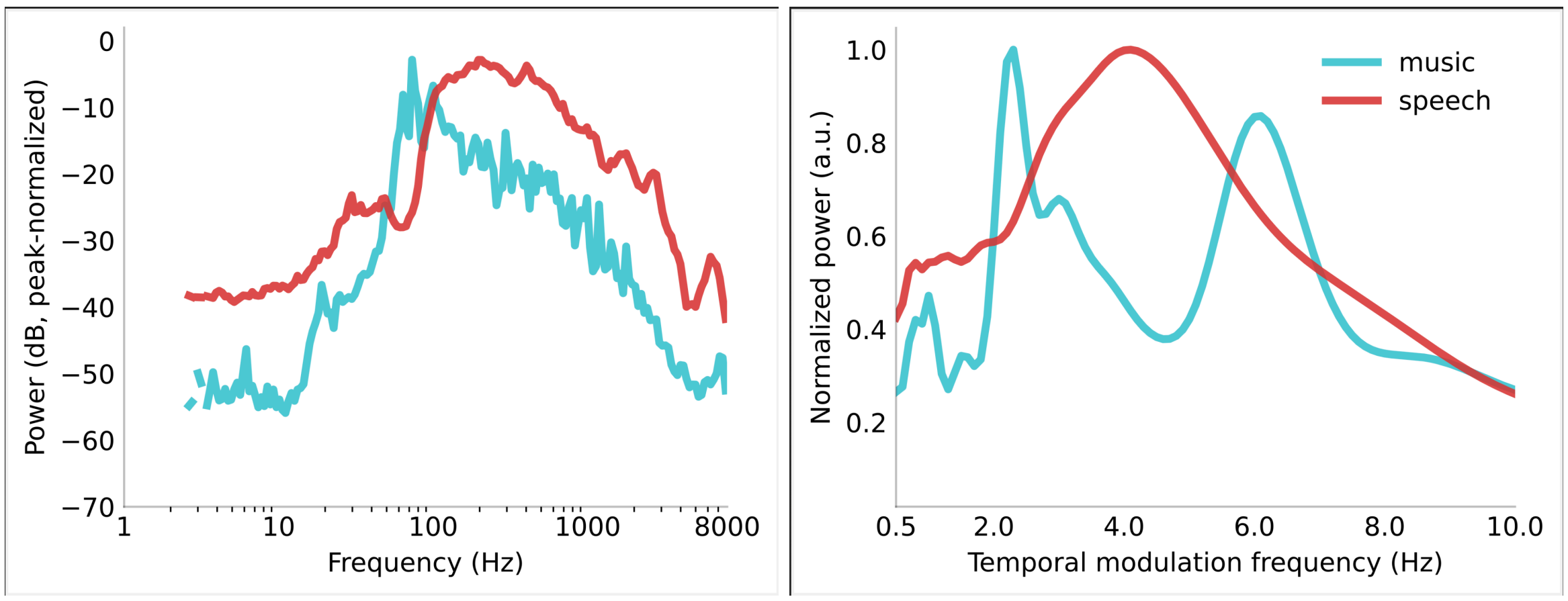


***Figure S5. (A)*** *Power spectrum (1–8000 Hz) of the continuous, 10-min-long speech and music stimuli, normalized to emphasize spectral shape rather than overall dB level.* ***(B)*** *Temporal modulation spectrum of the acoustic envelope (0.5–10 Hz) for the same stimuli, computed from the broadband envelope and normalized to highlight differences in modulation structure.*

## *Intersubject correlation additional analysis*

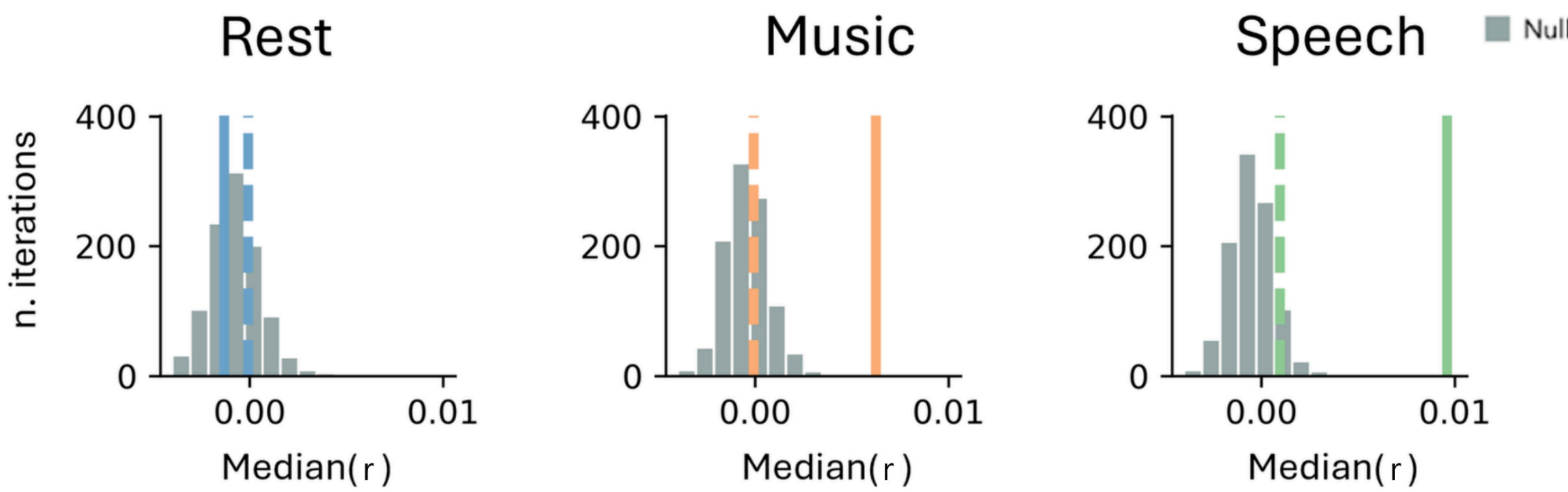


***Figure S6.*** *Median intersubject correlation compared to a null model. For both speech and music, the p-values—calculated as in Figure 1B—are p < 0.001. Similar to Fig. 1.B, the gray histogram represents the distribution of median intersubject correlation after random data shuffling. The blue, orange, and red lines correspond to the median of the observed intersubject correlations during rest, music, and speech, respectively. The dotted lines represent the intersubject correlation observed when considering only the lowest fluctuations in the high-gamma activity (see methods).*

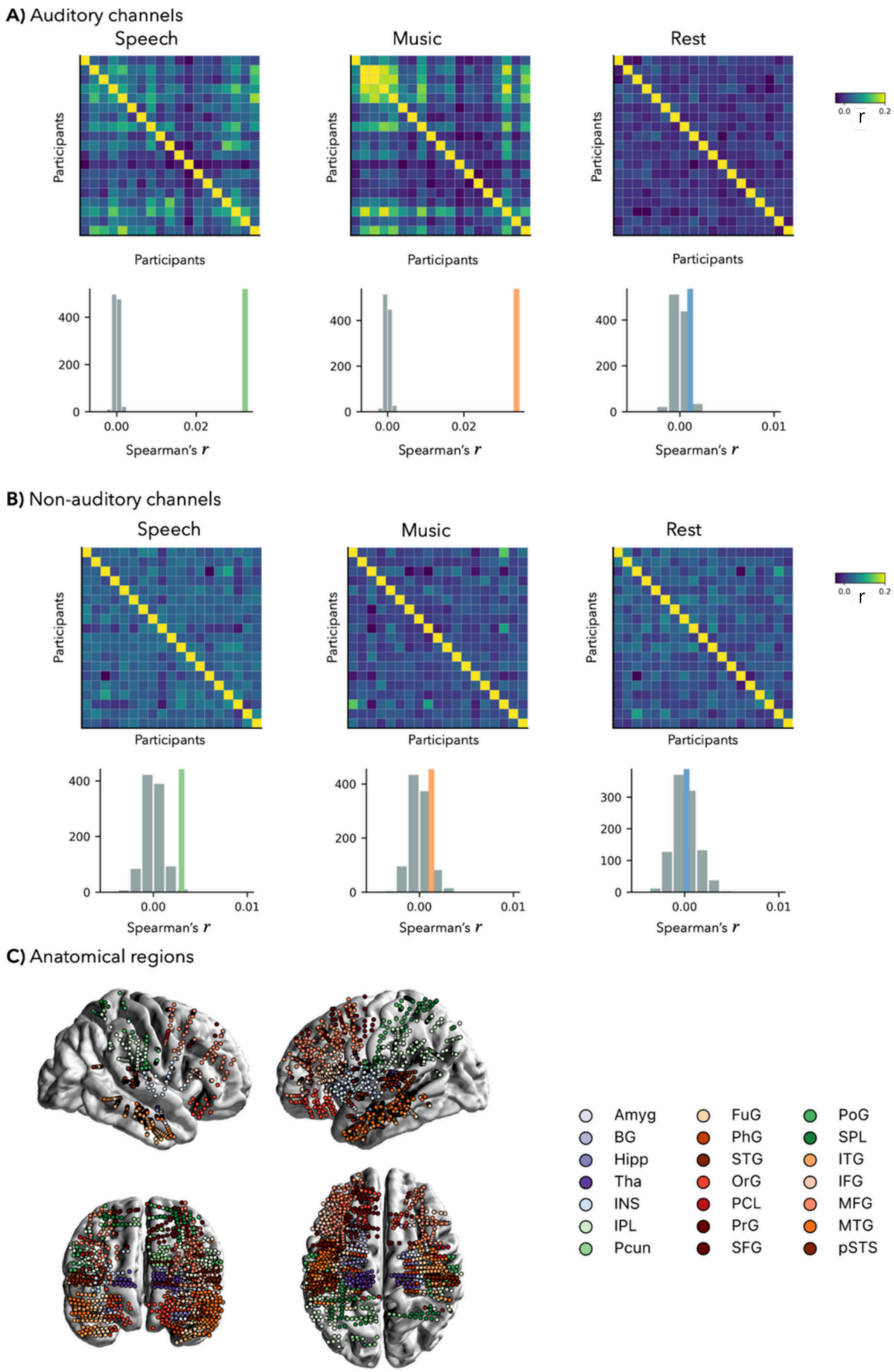


***Figure S7.*** *Inter-subject correlation of the activity profiles across conditions. Mean of the inter-subject correlation (coloured line), compared to a null model distribution (see Methods) for* ***(A)*** *auditory only electrodes (H) and* ***(B)*** *non-auditory only*

*electrodes (~H).* ***(C)*** *Anatomical regions defined according to the Brainnetome atlas (Fan et al., 2016). Abbreviations according to the Brainnetome atlas.*

## Activity engagement (AE) additional analysis

In the following section, we report additional analyses concerning AE. In principle, the difference in activity engagement between rest and speech (or music) can be driven either by higher AE during speech or music with respect to rest, or conversely by higher AE during rest (Fig. S9A). It is important to note that higher AE does not directly imply higher HFa; rather, it simply reflects a greater presence of either positive or negative bursts of activity in the HFa envelope time series. For this reason, in the main manuscript we report channels with significantly different involvement without distinguishing whether the involvement was higher during speech than rest, or vice versa. In this section, we present additional results related to these analyses.

First, we provide a table reporting the percentage of significant channels for all comparisons, separating cases in which one condition was higher than the other. We then show the topographies for all comparisons that are not included in the main manuscript. Specifically, we first report the direct comparison of speech versus rest, where channels showing higher values during speech are shown in green (orange for music), and channels showing higher values during rest are shown in blue. Second, we report the direct comparison of speech (green) versus music (orange) (Fig. S8B).

Finally, we repeated this analysis selecting only the time windows in which the intersubject correlation was higher than the maximum observed during rest (red dots in the bottom panels of Fig. 2B). The significant channels observed in this analysis are partially overlapping between speech and music, mostly within the auditory cortex, but also in associative temporal and frontal regions. These results indicate that the inter-subject correlation of APr during speech and music processing is driven by whole-brain network phenomena, not limited to local (auditory) processing.

| Direction | Significant | Total | Bonferroni % | Mean subject % | SD subject % | Uncorrected % |
|---|---|---|---|---|---|---|
| speech > music | 105 | 2472 | 4.2476 | 4.1663 | 6.8245 | 13.0259 |
| music > speech | 124 | 2472 | 5.0162 | 4.7788 | 5.781 | 16.2217 |
| speech > rest | 69 | 2472 | 2.7913 | 2.6804 | 3.5268 | 11.9337 |
| rest > speech | 167 | 2472 | 6.7557 | 6.7275 | 5.1595 | 20.7929 |
| music > rest | 43 | 2472 | 1.7395 | 1.5591 | 2.7799 | 9.3851 |
| rest > music | 106 | 2472 | 4.288 | 4.4563 | 3.9315 | 15.6553 |

**Table S1.** *Percentage of high activity engagement (high-AE) channels comparing all conditions (speech, music, and rest) in pairs. The percentages are computed over all the data points.*

While our analysis highlights differences in the networks engaged during speech and music listening, the percentage of channels for which the AE is significantly different when comparing speech versus music is relatively low (9.3% of the channels for the aggregated population; see Table.S1). This result supports previous evidence that speech and music processing mostly rely on shared resources (te Rietmolen et al., 2022). Similar low percentages are observed when comparing speech versus rest, and music versus rest (Table.S1), which suggests specific localized alterations of brain activity during speech/music processing compared to rest condition.

Finally, to test the complementarity between HFa and AE, we correlated the total amount of HFa recorded in each region with its activity engagement. The absence of a significant correlation highlights the complementary nature of these two approaches (Fig.S10).

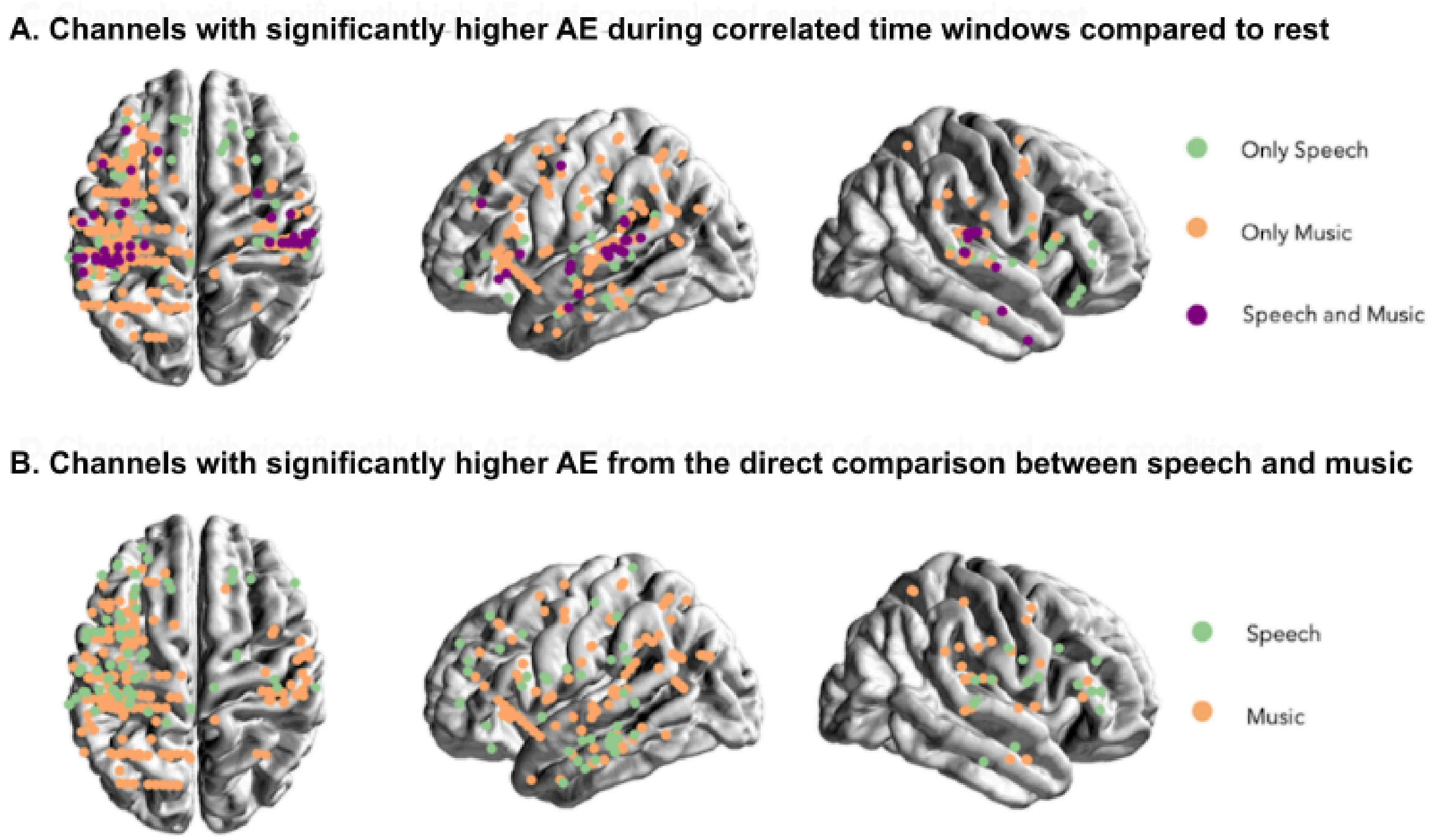


***Figure S8.*** *Panel **A)**, channels showing significantly higher activity engagement (AE) during speech and music compared to rest, restricted to time windows in which inter-subject correlation (ISC) during speech or music exceeded the maximum ISC observed during rest. Panel **B)**, channels exhibiting significantly greater AE during speech compared to music, and vice versa.*

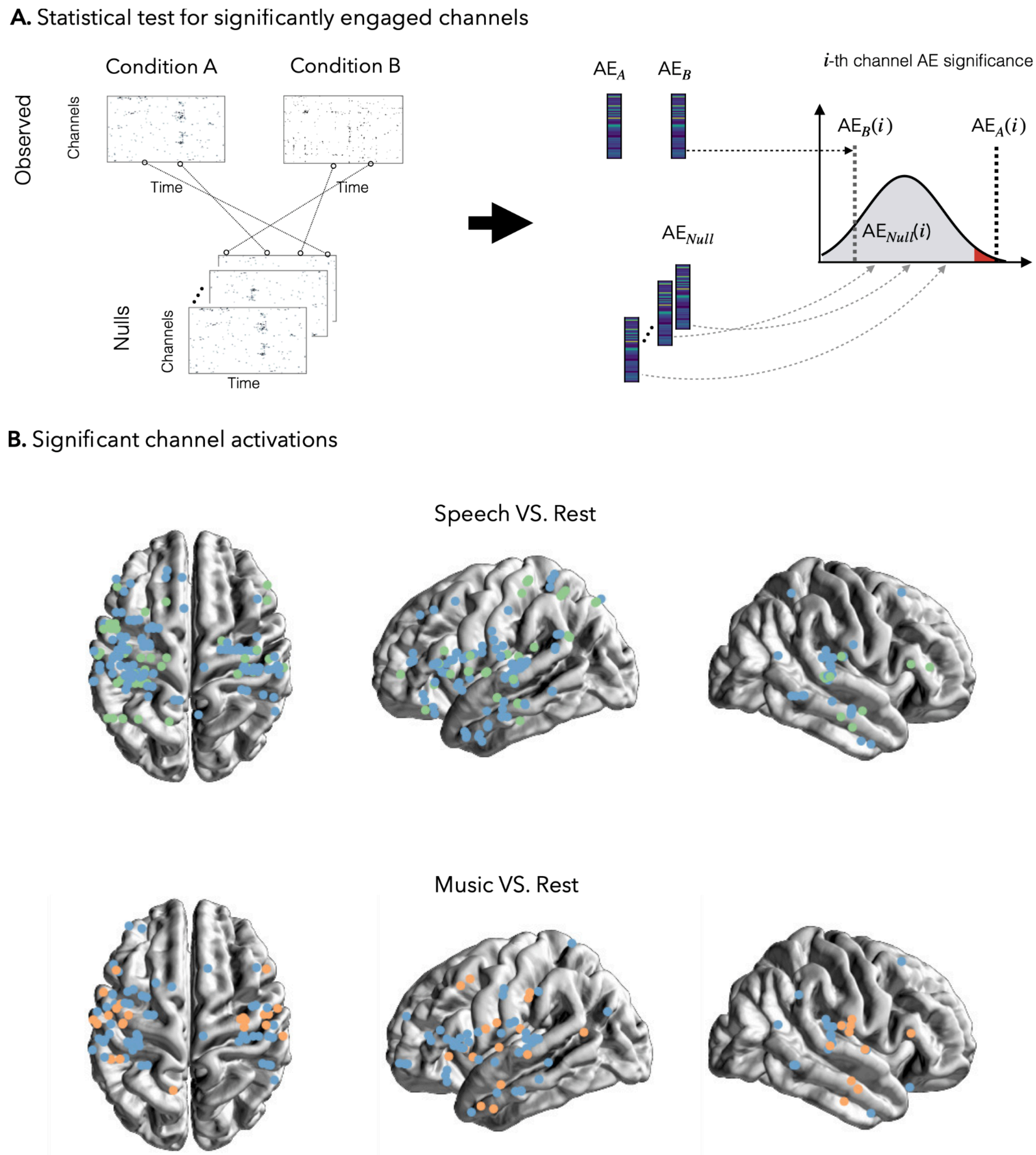


***Figure S9. (A)*** *The statistical test for comparing AE across conditions consists of 1) defining null binary sequences from the random combination of binary signals observed in conditions A and B (among speech, music, and rest); 2) extracting the AE for each null data and defining the (hypergeometric) null-distribution for each channel* $i$*; 3) compare the AE(*$i$*) observed in conditions A and B to the null-distribution of* $AE_{Null}(i)$*. Significantly engaged channels display AE(*$i$*) falling in the right tail of the null distribution (*$p<0.05$*, Bonferroni corrected for the number of channels).* ***(B)*** *Significantly engaged channels across all pairs of conditions (speech in green, music in orange, rest in blue) and across all participants.*

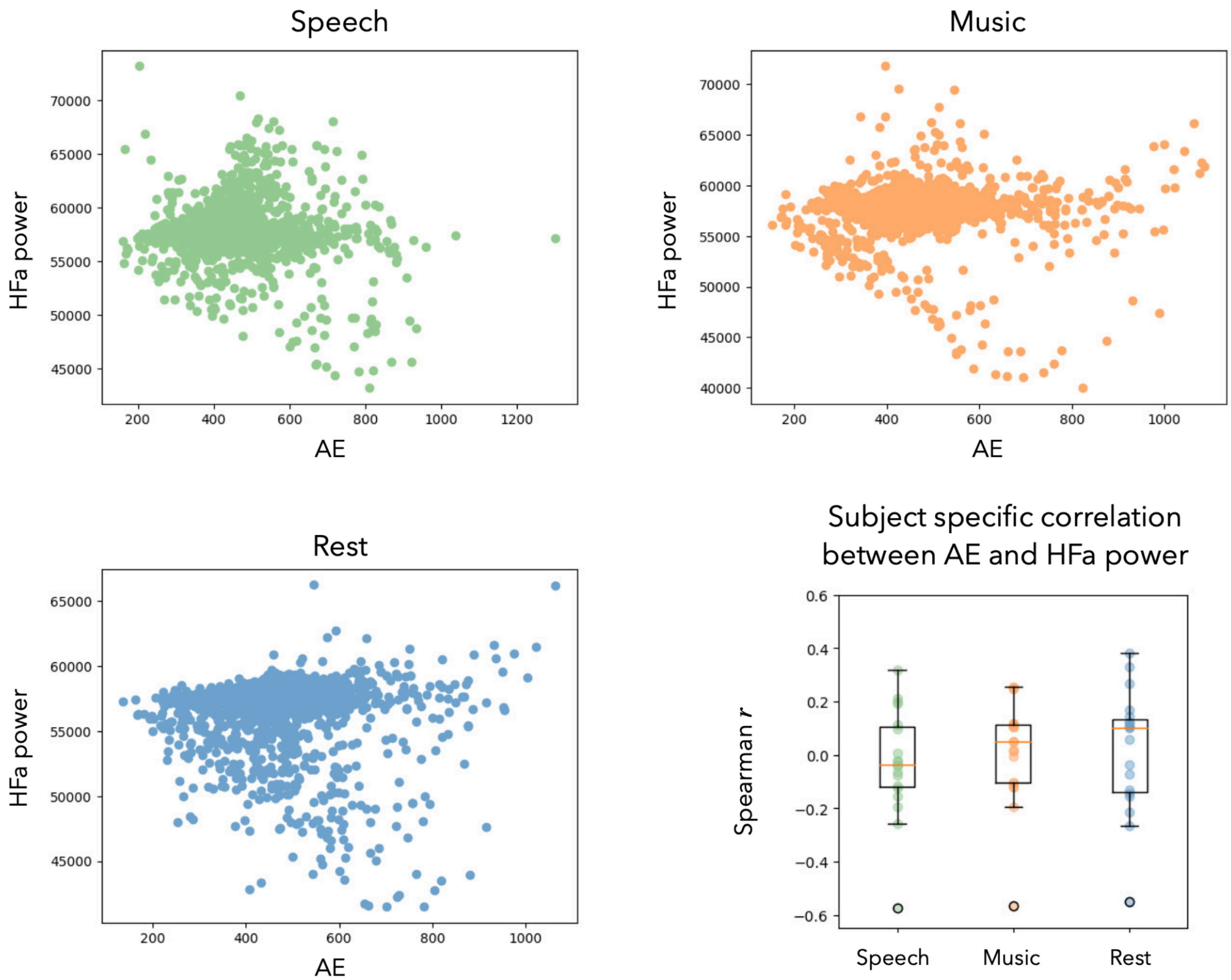


***Figure S10.*** *The three scatterplots show–for all channels present in our data –the HFa power, measured as the sum of the original filtered signals (not z-scored), and the AE, computed as the sum of the binarized (z-scored) signals. At the level of aggregate population these scatterplots show poor correlation values in all conditions (Speech, Spearman r = -0.00039, p=0.98; Music, Spearman r = -0.0558, p<0.05; Rest, Spearman r = -0.0765, p<0.05). When computed for each participant separately (bottom right panel), some correlation values between AE and HFa power were higher in absolute value, but the distribution of correlation values was not significantly deviating from zero (one sample t-test p>0.05).*

## Neuronal avalanches association to cortical timescales

In this section we show that the activity profile and AE measures obtained from the binarized activity can be associated with signals autocorrelation features, and interpreted as a proxy for the dynamics and topography of neuronal timescales.

In general, a characteristic timescale can be defined for a signal by studying the decay time of its auto-correlation function. Recent studies demonstrate that the brain's cortical timescales (e.g., measured by autocorrelation decay time) follow a smooth gradient spanning from posterior sensorimotor regions to anterior trans-modal hubs (Murray et al., 2014). Heterogeneous structural features such as brain microstructure, connectivity, cortical thickness, and gene expression, vary along the same axis (Fulcher, Murray, Zerbi, & Wang, 2019; Gao, van den Brink, Pfeffer, & Voytek, 2020), which points to a deep link between structure and function (Arbabshirani et al., 2014; Müller & Meisel, 2023). This hierarchical structure is preserved during the course of mammalian evolution, despite the increase of brain volume (Fulcher et al., 2019); it is characteristic of individual brains, and alterations in its organization lead to mental and neurological disease (Northoff, Sandsten, Nordgaard, Kjaer, & Parnas, 2021). However, differently than structural features, brain function is highly dynamic and the topography of timescales can vary over time both in rest (P Sorrentino et al., 2023), and task (Golesorkhi et al., 2022). For instance, visual stimuli presentation induces a decrease in the amplitude of the alpha band (8-12Hz), while mental calculation or working memory tasks increase the activity in this same frequency band (J. M. Palva et al., 2013; S. Palva & Palva, 2007).

While several measures for the signals timescales have been proposed (Müller & Meisel, 2023; P Sorrentino et al., 2023), here for illustrative purposes we evaluated the timescales as the area $\tau$ before the first minimum; Fig.S11.A, left). Even in the same recording channel, the decay of the auto-correlation can change when observed in different time windows (Fig.S11.A, right). To compare the timescales results to the neuronal avalanches results obtained in Fig.1, we first studied the time evolution of the timescale $\tau_i(t)$ for each channel $i$ (Fig.S11.B). Averaging across all time windows, we defined the Temporal Engagement (TE) showing that electrophysiological fluctuations at different channels are associated with different timescales on average (Fig.S11.B, right). Averaging across channels, we define the Temporal Profile (TP) which shows that the system's timescales can be shorter or longer depending on the observation window (Fig.S11.B, bottom). Notably, both the TE and the TP correlate with the activity engagement (AE) and activity profile (averaged in time windows to allow comparison), which were previously defined from the binarized activity (Fig.S11.C). This shows firstly that the fluctuations of the

system's time scales are captured by the avalanche approach, secondly that the spatial characterization of the timescales, e.g., in which brain regions are short/long timescales observed on average, depends on how many times the avalanches were passing from each brain region. Importantly, using the decay of the autocorrelation as a measure of dynamic timescales we obtained similar inter-subject correlation results as in the binary approach (Fig.S11.D). More specifically we have a significant inter-subject correlation during speech listening ($p < 0.001$) and during music listening ($p = 0.02$) and not significant during resting state ($p = 0.877$). The significance of the inter-subject correlation between TPs is assessed by shifting 1000 iterations as for the activity profiles.

Since we show that both the time profile of binary activations (activity profile) and the spatial topography of activations (Activity Engagement AE) are highly correlated with related temporal (Time Profile TP) and spatial (Time Engagement TE) metrics obtained from spectral features, the presented metrics could be used in the future to analyze timescale dynamics at a fine-grained temporal resolution and low computational cost. Overall, the observation of increased neuronal avalanche activity in association with slower neuronal timescales provide evidence for neuronal avalanches being involved in slow top-down processing of information.

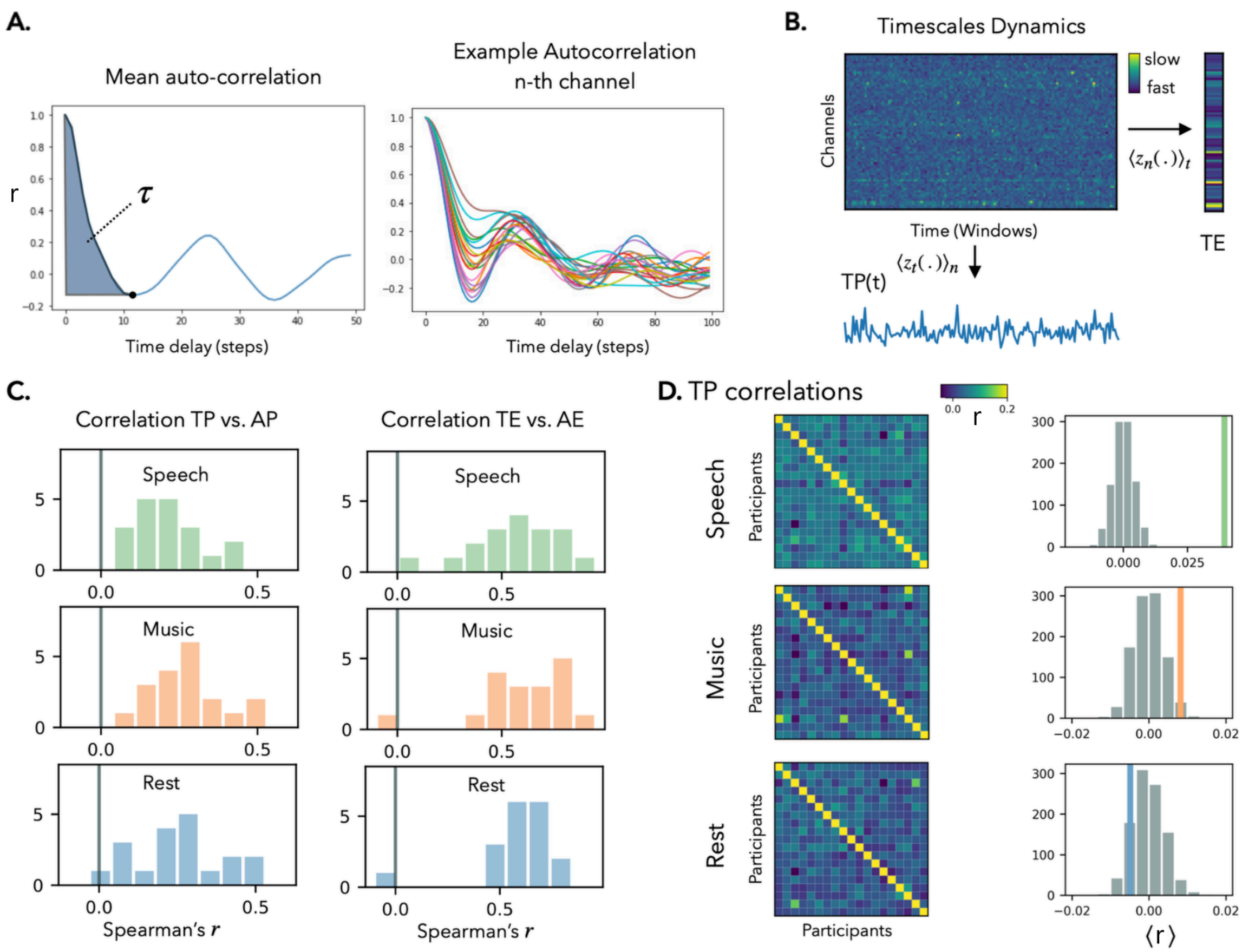


**Figure S11.** ***A, left)*** *A timescale* τ *for a brain signal was defined as the area under the autocorrelation plot, between 0 and the first minimum. For each channel, this plot can be obtained and* τ *can be computed in different time windows.* ***A, right)*** *In a single channel, the autocorrelation decay time can vary over time (each color represents different time windows).* ***B)*** *For each channel the timescales are computed as explained in (A) in windows of 150 time steps (3 seconds) and their evolution in time is investigated using a sliding window approach. The temporal evolution of the timescales is plotted in channels x time windows fashion. The Temporal Profile (TP) and Temporal Engagement (TE), computed by summing the timescales dynamics matrix across channels and time, respectively, are depicted below and on the right side of the matrix.* ***(C)*** *The distribution of the correlation values between the activity profiles and TPs (left side), and between the AEs and TEs (right side) in each condition.* ***D, left)*** *Inter-subject correlation matrices computed on the TPs.* ***D, right)*** *the comparison with the null model, computed randomly by time-shifting the TPs is shown and a statistic is computed.*

## Additional safety analysis for intersubject correlation

We repeated the inter-subject correlation analyses presented in Figure 1 to assess whether the observed trends could be driven by the age distribution of the participants, in particular by the inclusion of the 8-year-old subject, the other participants being all above 17 years old. We found that the results remained significant after excluding this participant (Fig. S12), indicating that the main findings are robust to age-related effects. To demonstrate that the inter-subject correlation (Figure 1) result is consistent across participants, we computed, for each individual, the mean correlation between their activity profile and that of all other participants (Figure S13). We found that 17 out of 19 participants exhibited higher average intersubject correlation during speech and music compared to rest, indicating that the effect is robust at the individual level rather than driven by a subset of participants. This additional analysis suggests that the observed effect is not attributable to participant-specific factors that vary across the population (e.g., age or listening modality), but instead reflects a consistent phenomenon across the cohort.

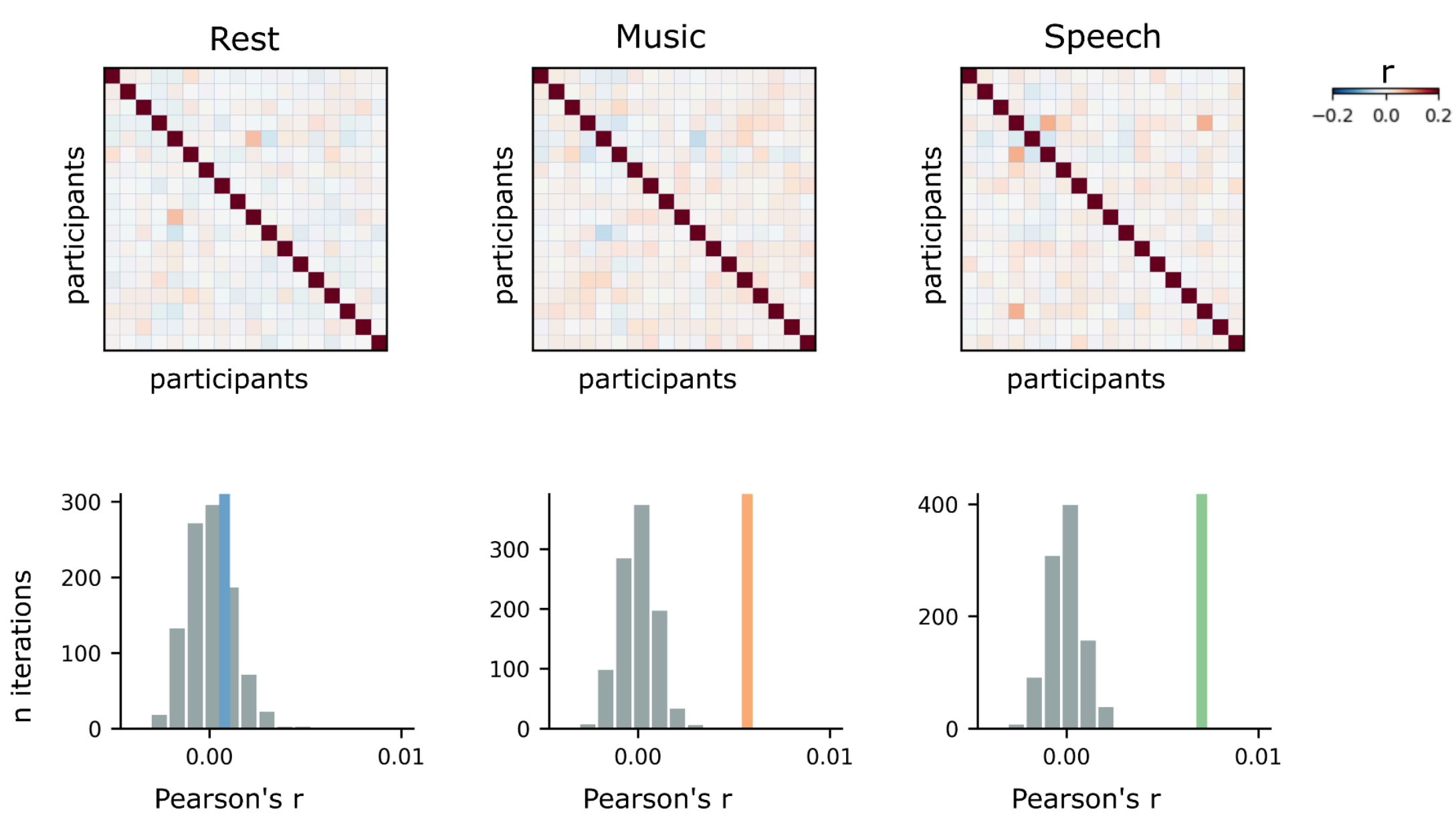


***Figure S12.*** *Replication of figure 1b, excluding the non-adult participant (8 years old).* ***(top)*** *Inter-subject (N = 18) correlation matrix for the three conditions (rest, music, speech). Each point of the matrix represents the correlation value between the activity profiles of two participants.* ***(bottom)*** *Mean of the inter-subject correlation (colored line) compared to a null model distribution (see Methods; *p<0.001).*

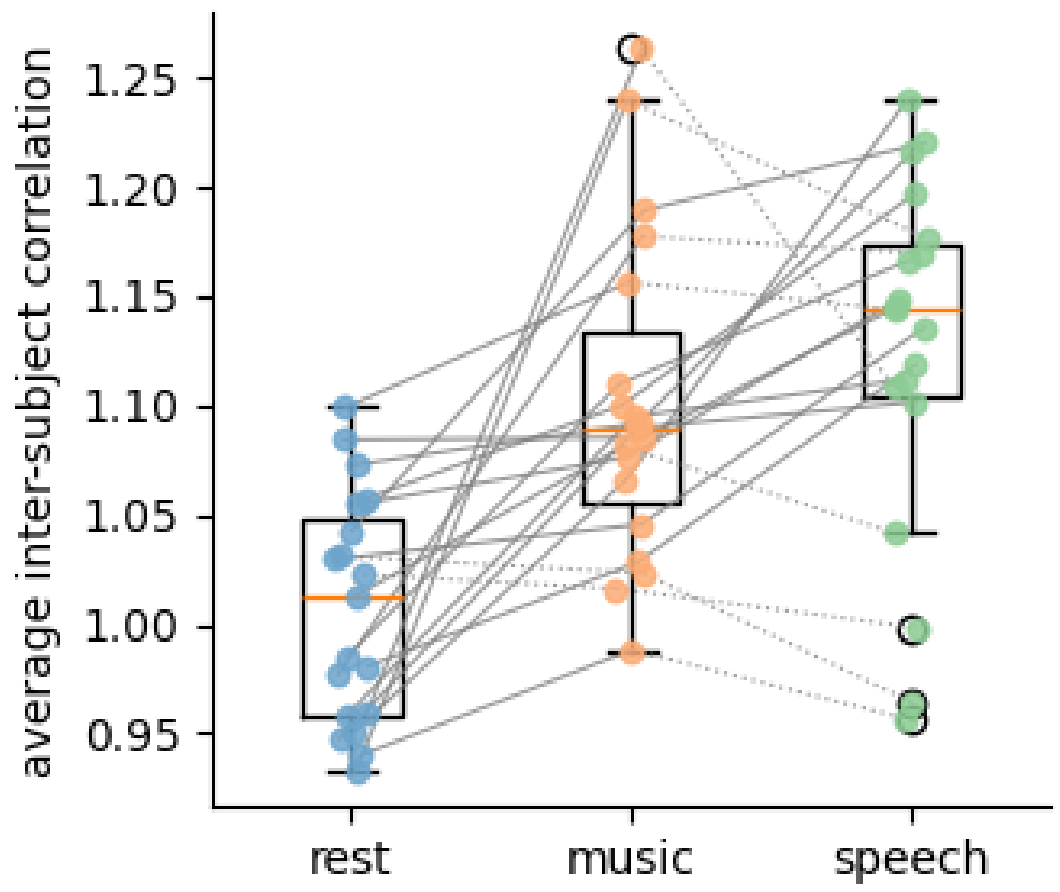


***Figure S13.*** *For each participant and condition (speech, music, rest), we computed the mean Pearson correlation between that participant's activity profile and the corresponding profiles of all other participants. Each dot represents one participant, and colors denote condition. Higher values indicate greater similarity to the activity patterns of other participants.*
